# Neurological tissue dissection techniques in mouse models for reproducible scientific results: Brain, spinal cord, cerebrospinal fluid, and sciatic nerve

**DOI:** 10.1371/journal.pone.0358771

**Published:** 2026-10-07

**Authors:** Lilia Crew, Alyssa Seerley, Serena McElroy, Andrea Grindeland Panter

**Affiliations:** 1 Touro University College of Osteopathic Medicine, Great Falls, Montana, United States of America; 2 Weissman Hood Institute at Touro University; McLaughlin Research Institute, Great Falls, Montana, United States of America; Hamad Medical Corporation, QATAR

## Abstract

Biomedical research studies, specifically regarding human neurodegenerative diseases, are bound by ethical challenges, and have limited diagnostic and treatment options. Transgenic mouse models provide the opportunity to conduct feasible and practical research with the ability to precisely define the progression of neurodegenerative disease over a well-controlled dosage and timeline. The use of transgenic mouse models has been extensive and is critical to advancing research in many ways, including understanding brain morphology and general tissue changes caused by neurological diseases. Often, these studies require specific brain regions or other neurological tissues which may be difficult to obtain. Unfortunately, specific extraction and dissection protocols are few and far between, leading to inconsistent results and a lack of reproducibility. A well-defined protocol, for dissection and extraction, is instrumental for acquisition of reproducible experimental results. Five mouse-specific protocols are described here: brain extraction, brain microdissection, spinal cord extrusion, cerebral spinal fluid collection, and sciatic nerve dissection. Each protocol was completed under biosafety level 2 (BSL-2) guidelines, similar to the sterility precautions required in human surgery. Each protocol also includes a required materials list that defines proper instruments and usage. The protocols were refined based on feedback from numerous research studies in transcriptomics and pharmaceutical development. These protocols minimize tissue damage, increase dissection accuracy, and increase reproducibility of results while developing skills directly transferable to clinical settings. This study provides well-defined, succinct, accessible protocols that support advancements in many cross-disciplinary areas.

## Introduction

Biomedical investigations into human neurodegenerative diseases face complex challenges and limitations of diagnostic and therapeutic modalities. Animal models have been used to study neurodegenerative diseases since the late 1950s, leading to substantial breakthroughs in understanding of neurodegeneration [1]. Mouse models have several advantages in modeling neurodegenerative diseases due to the remarkable similarity of the laboratory mouse, *Mus musculus*, and human genomes; indeed, approximately 90% of mouse and human genomes can be divided into regions of conserved synteny [2]. Humans and mice also share numerous developmental milestones at both the structural and cellular levels, as well as similar age-related behavioral traits [3]. Additionally, studies that compared transcriptome networks in human and mouse brains have demonstrated significant conservation of gene expression and connectivity, as well as the preservation of network modules [3].

Tissues from transgenic mouse models are readily obtainable and frequently recapitulate key aspects of neurodegenerative diseases while enabling design of experiments which are statistically powered under controlled laboratory conditions, contributing to higher quality data generation [4–11].

For example, the brain can be extracted and subsequently dissected in disease studies that focus on specific regions of the brain. Applications include identification of biomarkers found in the brain and cerebrospinal fluid (CSF) that have proven to be necessary in studies of various neurodegenerative disease models aimed at development of diagnostic tests and/or pharmaceutical treatments [12,13]

Despite the value of neurological tissues from animal models for increased understanding of neurodegeneration, dissecting specific tissues often presents a multitude of challenges for researchers including maintenance of tissue integrity, efficient dissection time frames, obtaining specific tissues without contamination from other tissues, equipment limitations, and highly specialized training requirements. Brain tissue is extremely delicate and malleable; improper handling and omission of care can easily alter or destroy target structures. This may result in compromised downstream analysis of histology and molecular assays and difficulty in replication [7]. Prompt extraction and proper fixation of tissues can help to eliminate ischemia and tissue degradation in neurological tissue studies and avoid inconsistencies in data and a lack of reproducibility across studies [7,14].

Although references can be found regarding the techniques highlighted [7],[14–31], our manuscript offers a practical collection of neurologic tissue microdissection techniques that are often used together with descriptions applicable for efficient workflow. The techniques are specific for the laboratory mouse and are unique in methods from other resources, providing more accessible alternatives. Previous resources are frequently constrained by limited accessibility, such as high-cost subscriptions and specialized equipment needs, or by a steep learning curve that can be difficult for novice scientists to navigate. A summary of the literature is displayed in Table 1. Below, are brief descriptions of how each specific method differs from previous resources.

**Table 1:** Existing resources and literature for each method.

Method	Title of Manuscript
CSF collection	An improved method for collection of cerebrospinal fluid from anesthetized mice [18]
	A protocol for collection and infusion of cerebrospinal fluid in mice [20]
	A technique for serial collection of cerebrospinal fluid from the cisterna magna in mouse [15]
	Anatomy-oriented stereotactic approach to cerebrospinal fluid collection in mice [19]
Brain dissection	Dissection of different areas from mouse hippocampus [14]
	Dissection of mouse hippocampus with its dorsal, intermediate and ventral subdivisions combined with molecular validation [23]
	Dissection of rodent brain regions [24]
	Dissection of rodent brain regions: guided free-hand slicing and dissection of frozen tissue [25]
	Microdissection of mouse brain into functionally and anatomically different regions [7]
	Protocol to dissect and dissociate the mouse brainstem for single-cell RNA-seq applications [26]
	Rodent brain extraction and dissection: A comprehensive approach [21]
Sciatic nerve collection	Denervation of mouse lower hind limb by sciatic and femoral nerve transection [27]
	Harvesting the maximum length of sciatic nerve from adult mice: a step-by-step approach [16]
	Protocol for isolating and processing mouse sciatic nerve fibers for confocal immunohistochemistry [22]
Spinal cord	A simple, step-by-step dissection protocol for the rapid isolation of mouse dorsal root ganglia [28]
	Primary culture of mouse neurons from the spinal cord dorsal horn [29]
	Protocol for dissection and culture of murine dorsal root ganglia neurons to study neuropeptide release [30]
	Protocol to prepare mouse spinal cord for patch-clamp and histology experiments [31]
	Hydraulic extrusion of the spinal cord and isolation of dorsal root ganglia in rodents [17]

Obtaining CSF is exceedingly difficult, typically requiring specialized equipment [15,18–20] that is costly and oftentimes too large for biohazardous work inside a biosafety cabinet. Our technique describes a simplified protocol that may be effectively performed in a biosafety cabinet with little equipment.

The brain dissection and brain region microdissection methods reported here are mouse-specific techniques and differ from the methods previously reported [7,14,21,23–26], offering the reader additional techniques that are simpler to perform effectively.

Previous resources providing methods for obtaining sciatic nerve samples exist [16,22,27]; however, ours is a simplified method with clear images, instructions for proper handling to minimize damage, and focus on dissection of healthy nerve tissue as opposed to many references which report injuries or surgical techniques to study damage of the sciatic.

Many techniques exist for spinal cord dissection in the mouse [28–31], however, our hydropulsion technique has many advantages such as simplicity of procedure and preservation of tissue over other methods, many of which manually dissect out the spinal cord from the vertebrae. Our hydrostatic method differs somewhat from the other reference [17] despite using a similar technique and includes additional information such ensuring the correct fit of the syringe occurs and sectioning into specific spinal regions.

While the methods described below involve subtle refinements to existing protocols, they offer practical advantages for researchers with limited specialized equipment or dissection experience. This accessibility fosters greater experimental consistency and enhances scientific reproducibility [6].

## Materials and methods

### Animals

The mice included in this study were housed in the Weissman Hood Institute at Touro University; McLaughlin Research Institute’s Animal Resource Center, which is an all-mouse facility and is accredited by the Association for Assessment and Accreditation of Laboratory Animal Care International (AAALAC). All animal studies were conducted in accordance with the US National Research Council’s Guide for the Care and Use of Laboratory Animals, the US Public Health Service’s Policy on Humane Care and Use of Laboratory Animals, and the Guide for the Care and Use of Laboratory Animals. The animal procedures and studies were reviewed and approved by the McLaughlin Research Institute’s Institutional Animal Care and Use Committee (IACUC) under protocol number (2023-AG-100) and biosafety protocol number (2022-AG-IBC05), approved for the mice and procedures.

Three lines of mice were used for the study: FVB/NJ, C57BL/6J and Cer-PrP-225SS (Tg1536) [8,32]. Wild-type mice on the FVB/NJ and C57BL/6J background strain were used for the sections with images demonstrating methods for normal, non-diseased tissue dissection techniques, n = 3 (Fig.4-13). Samples demonstrating techniques that were performed under biosafety precautions were obtained from chronic wasting disease (CWD) inoculated (n = 8, 4 female and 4 male) or normal brain homogenate (NBH) inoculated (n = 8, 4 female and 4 male) mice from the transgenic mouse line Cer-PrP-225SS (Tg1536), which expresses cervid PRNP on a mouse PRNP knockout background. This group was included as a prion-positive cohort to demonstrate quality and repeatability of CSF biomarkers and histological tissue specimens in brain and spinal cord despite additional biosafety precautions such as biosafety cabinets and additional personal protective equipment (PPE) (Fig 14 and 15A&B). These mice were all at CWD endpoint ages, 9.4 months of age, total N = 20.

### CSF biomarker analysis

The Mesoscale Discovery System (MSD) MESO QuickPlex Q 60MM with the S-PLEX Neurology Panel 1 kit (#K15639S, Rockville, MD) was used to detect t-Tau in 20-fold diluted CSF in prion diseased transgenic mouse models. Product protocols were followed to improve accuracy and repeatability of results and duplicate samples were performed. Calibration procedures followed the manufacturer’s recommendations, with standard curves generated separately for each biomarker. Methodical Mind Enterprise Analysis Software (Meso Scale Discovery, Rockville, MD) was used for quantitative data analysis. Individual CSF concentration datapoints were calculated by averaging the two replicates.

### Surgery preparation

Before beginning any of these procedures, implement the appropriate biosafety level methods for proper safety and decontamination purposes, and familiarize yourself with an overview of procedural order (Fig 1**)**. All instruments must be gathered as shown in Fig 2 and sterilized in advance. Instruments should be sharp and good quality. CSF collection is the only procedure in this report performed under terminal anesthesia. All other protocols are performed post-euthanasia. Administer the IACUC approved anesthesia if CSF collection will be performed. Ensure that the appropriate level of anesthesia is reached by lack of deep pain reflex and minimal respiratory response to a toe pinch with a hemostat. Following CSF collection, euthanize the mouse in accordance with AVMA and IACUC guidelines.

**Fig 1:**
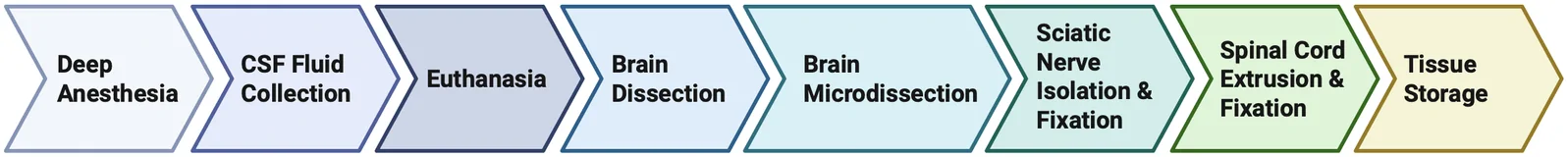
Experimental Workflow for CNS & PNS Dissection. Graphical overview of the sequential workflow highlighting the key stages of the dissection protocol. While shown in sequence, the protocol is modular, allowing researchers to adapt or omit steps based on specific experimental needs. Created in BioRender. https://BioRender.com/rfi9rxe.

**Fig 2:**
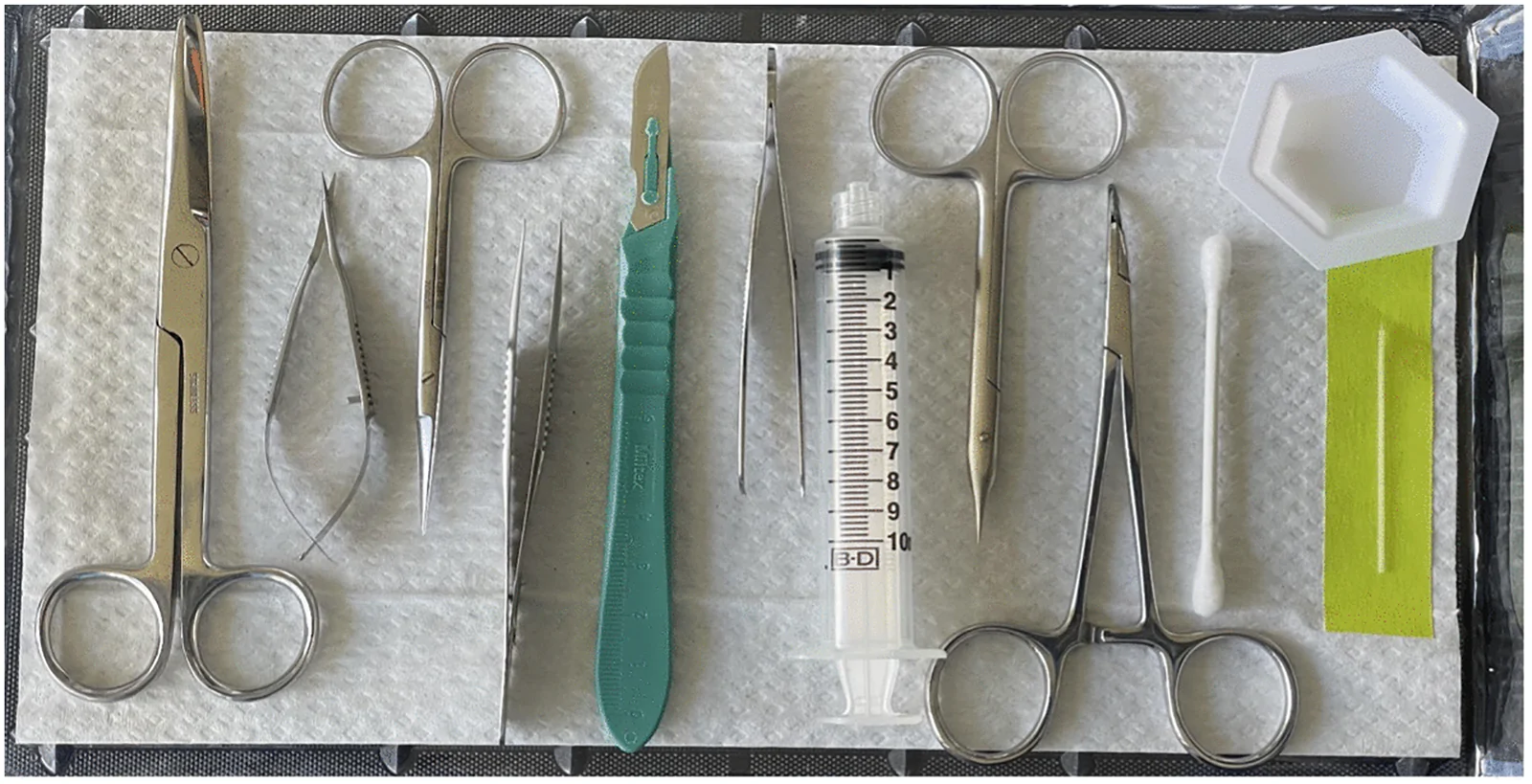
Dissection Instruments and supplies useful in microdissections. [A] Large Scissors; [B] Ophthalmic Microdissection Scissors; [C] Small Scissors; [D] Fine Tip Forceps; [E] Scalpel (#10 blade); [F] Curved Forceps; [G] Luer Lock Syringe (10mL); [H] Small Fine Tip Microdissecting Scissors; [I] Curved Hemostat, [J] Cotton Swab, [K] Weigh Boat; **[L]** Capillary Tube (on yellow tape). Not pictured: 70% EtOH, Petri Dish, PBS (1X concentration), Formalin (10%), Dissection Pins.

### Capillary tube for CSF collection

Using a micropipette puller, make a needle from a 10 cm length capillary tube made of polished borosilicate glass with filament, size O.D.:1.0 mm I.D.: 0.78 mm. The end of the needle will need to be cut to a diameter of 0.2 mm, as the larger diameter will allow the CSF to flow into the tube by capillary action, see Fig 3.

**Fig 3:**
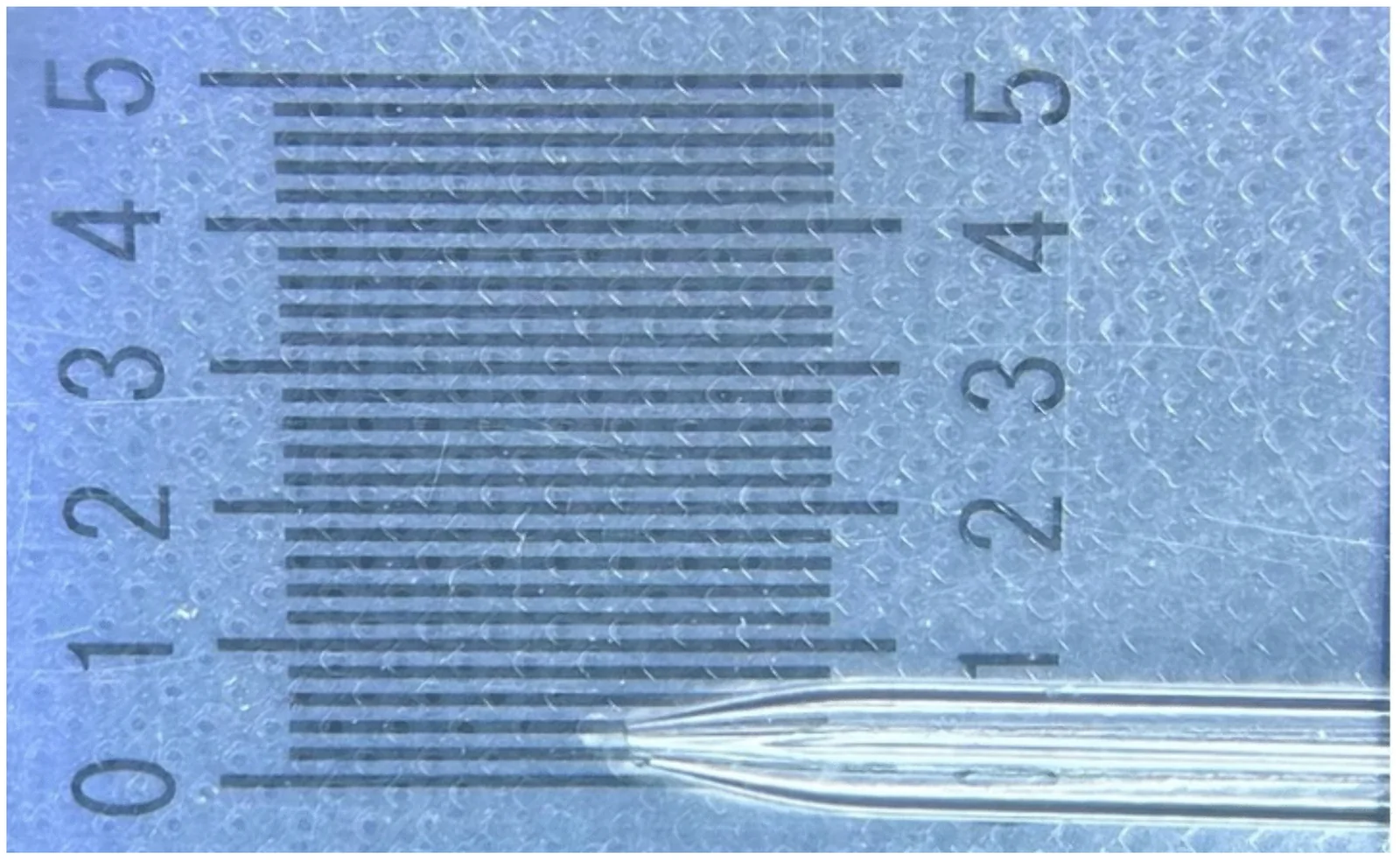
Capillary tube recommendations. Borosilicate glass with filament [O.D.: 1.0 mm, I.D.: 0.78 mm, 10 cm length] capillary tube pulled into a needle and cut leaving a 0.2 mm diameter opening for puncture of the arachidonic membrane.

### Terminal cerebral spinal fluid (CSF) collection

The following protocol described in this peer-reviewed article is published on protocols.io (https://dx.doi.org/10.17504/protocols.io.36wgqpnwxvk5/v2) and is included for printing purposes as S1 File.

1. Apply 70% ethanol (EtOH) to the surgical area to wet down the hair of the mouse at the nape of the neck.
2. Place the mouse in prone or sternal recumbency position. Using a #10 scalpel blade, create a midline skin incision extending from the occiput to the level of the second cervical vertebra (C2). If needed, gently flex and extend the skull to identify the precise location of the occiput before making the incision.
3. Maintain the mouse in sternal recumbency with the skull in flexion. Using sharp dissection, incise along the midline between the cervical musculature from the occiput to C2. The cervical muscles rhomboideus cervicis, the cervical portion of the trapezius, splenius capitis, semispinalis capitis, and erector spinae muscles should be separated at the midline to expose the cisterna magna. Deeper muscles that are difficult to visualize grossly may be separated under the dissecting microscope in Step 6 using blunt dissection techniques.
4. Place the ventral surface of the thorax of the mouse on the weight boat under the microscope.
5. Stabilize the mouse, ensuring that the skull is hyper flexed for maximum access to the cisterna magna, see Fig 4.
6. Bluntly dissect and separate at midline the interior muscles remaining from step 3 down to the cisterna magna to visualize a transparent membrane. This is the arachnoid membrane covering the cisterna magna, which is a large pocket of CSF within the subarachnoid space. If hemorrhage from the musculature occurs, blot it with a cotton swab prior to puncturing the arachnoid membrane as it is critical to avoid blood contamination in the CSF, see Fig 5.
7. Once the arachnoid membrane is visualized, use small thumb forceps to move the muscles laterally if needed for complete visualization.
8. With the capillary tube, puncture the arachnoid membrane at a 45° angle, using a gentle spinning motion.
9. Allow CSF to flow into the tube by capillary action. The approximate amount of CSF collected is between 4–10 µL, see Fig 6.
10. Place the blunt end of the capillary tube into the rubber bulb capillary dispenser instrument and expel the sample into the desired sample tube, see Fig 7.

**Fig 4:**
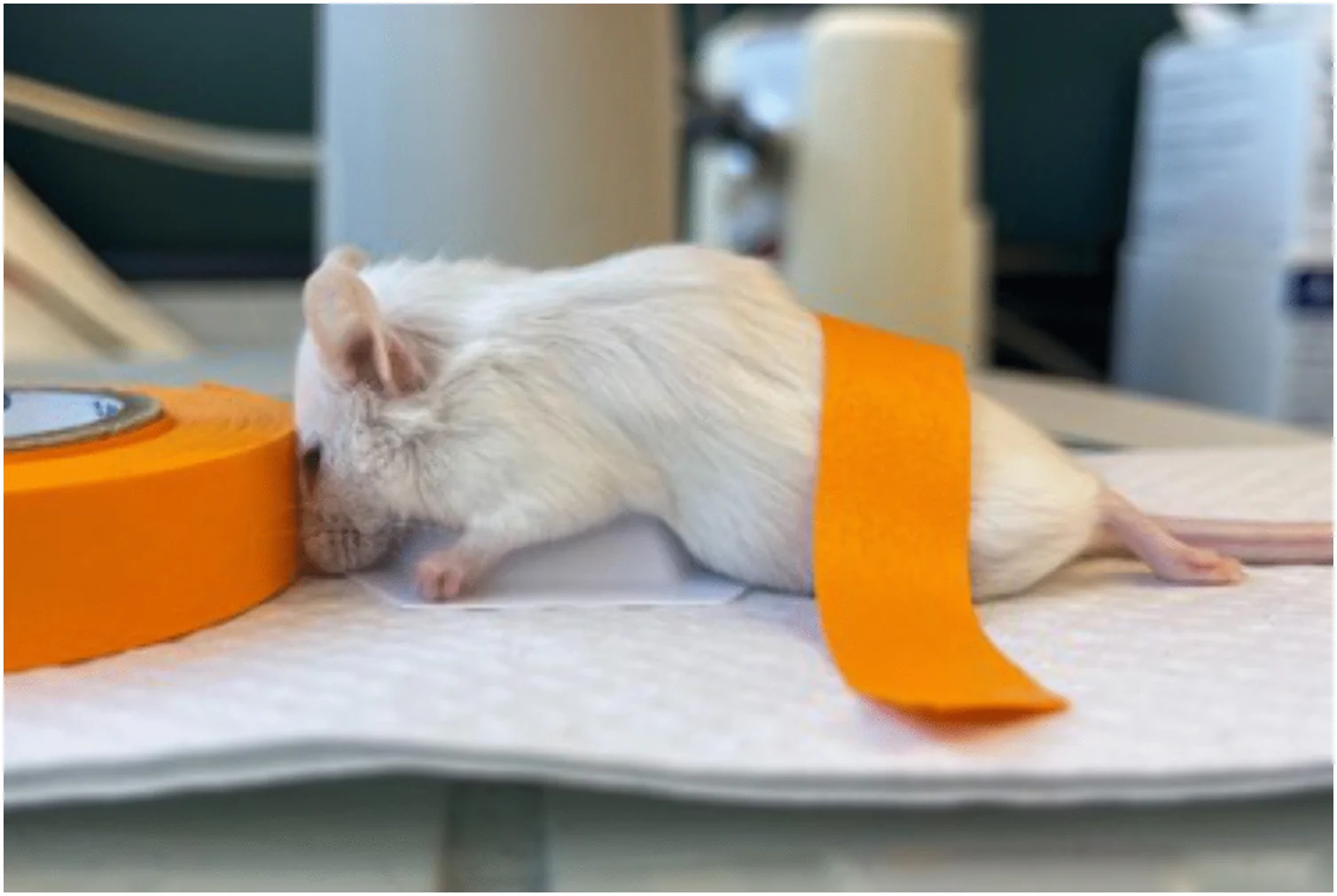
Mouse Positioning to obtain CSF. Anesthetized mouse positioned for cerebrospinal fluid (CSF) collection, demonstrating hyperflexion in the cranial-occipital region. The mouse is secured with orange tape to maintain the necessary posture for effective CSF extraction and hyperflexion is induced via a weigh boat and tape roll or any object that will hold the correct stabilization and placement of the head.

**Fig 5:**
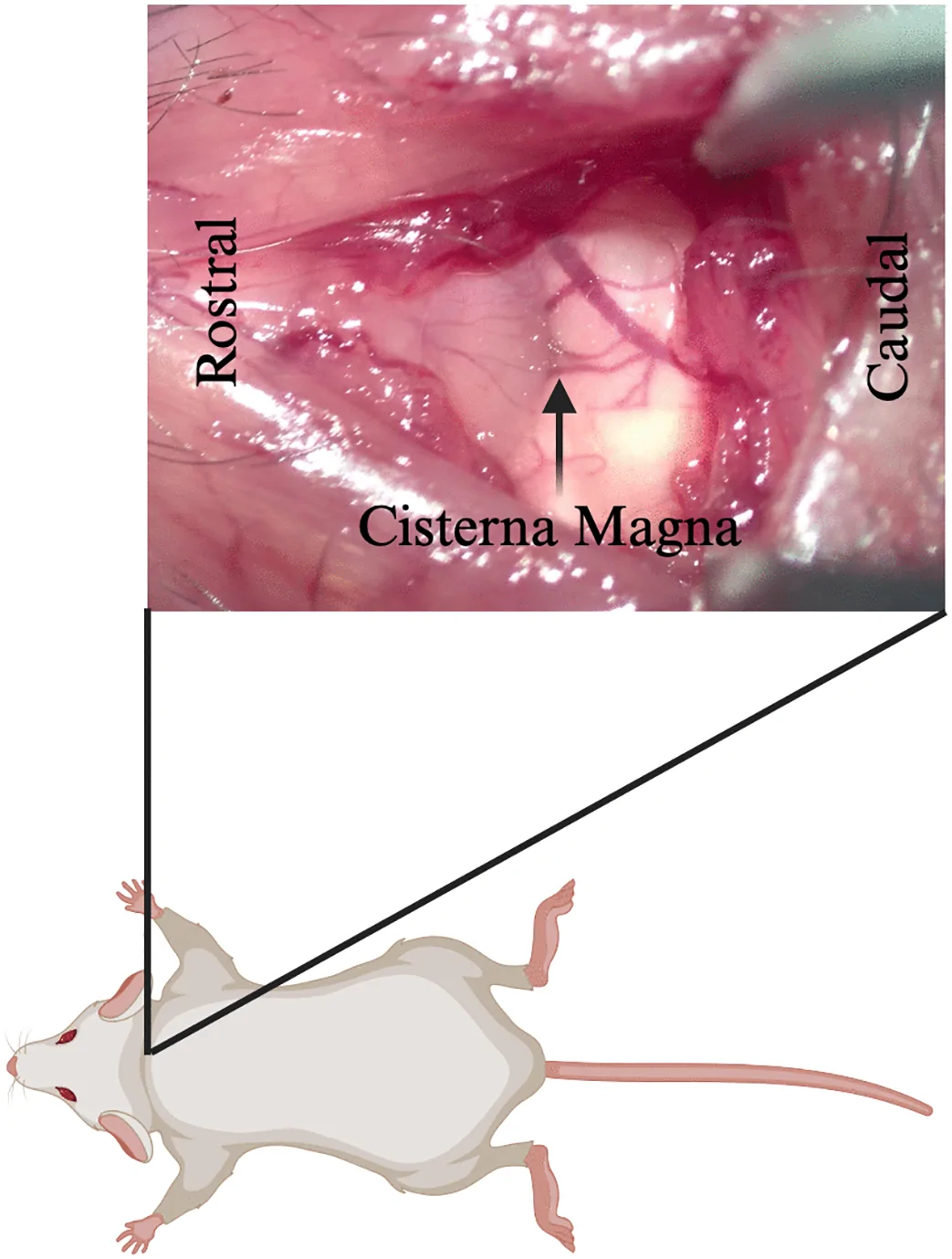
Visualization of cisterna magna. Created in BioRender. https://BioRender.com/tbp6pty.

**Fig 6:**
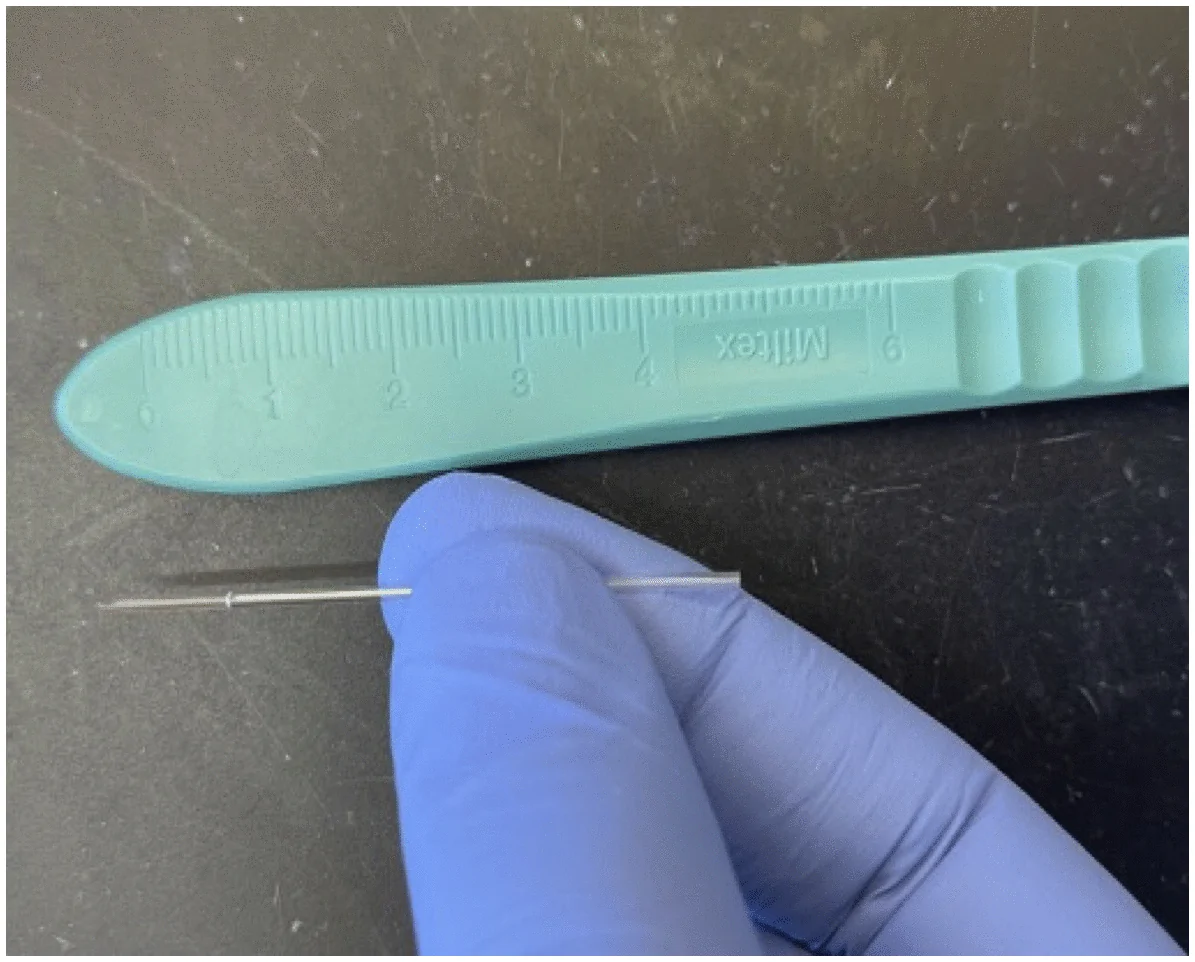
Capillary tube containing CSF.

**Fig 7:**
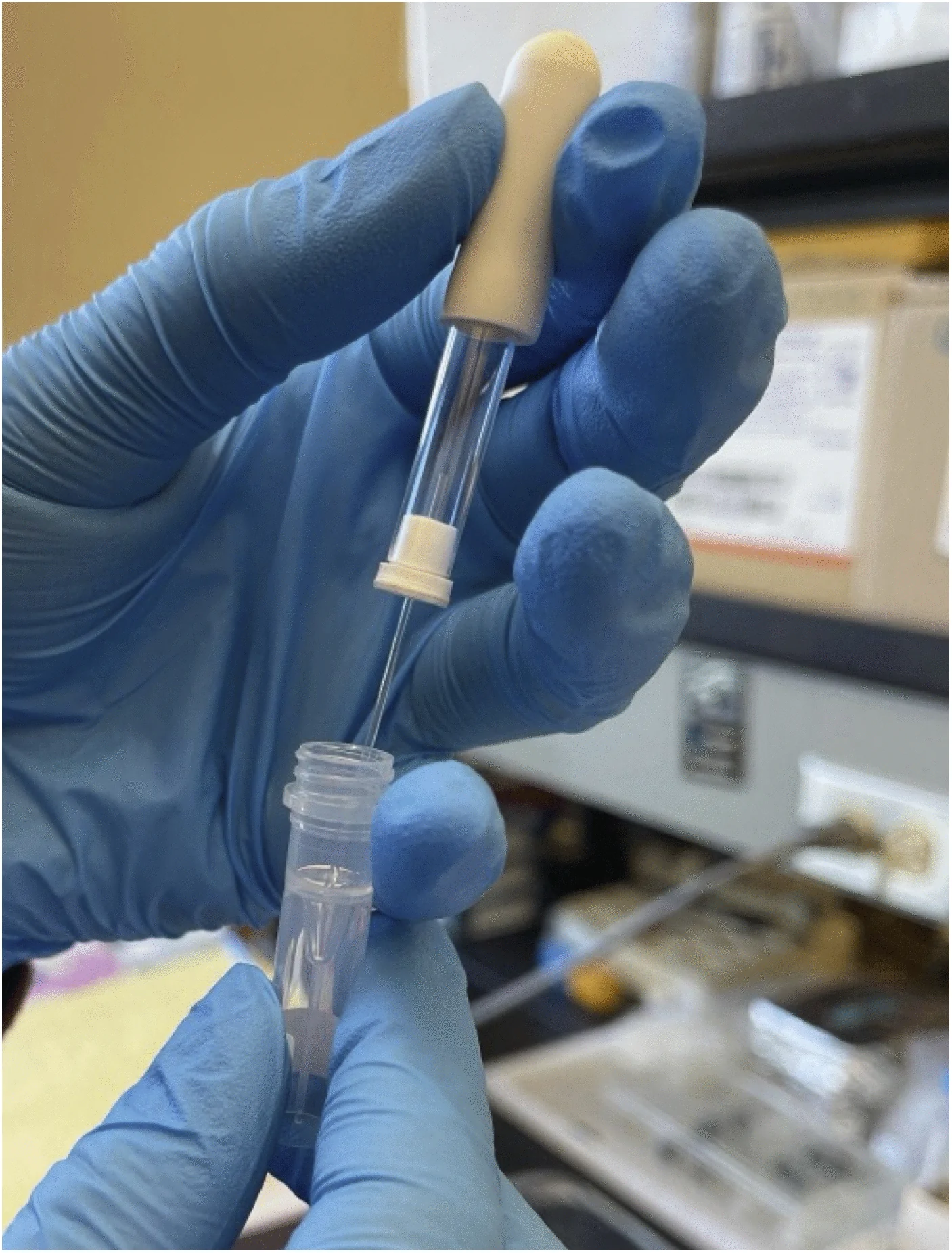
Transference of Collected CSF. Using a rubber bulb capillary dispenser into a storage vial in a laboratory setting. This process ensures the integrity and sterility of the sample for subsequent analyses.

### Brain extraction

The following protocol described in this peer-reviewed article is published on protocols.io (https://dx.doi.org/10.17504/protocols.io.6qpvrbqy3lmk/v2) and is included for printing purposes as S2 File.

1. Place the mouse in a ventral position.
2. Spray the cranium with 70% EtOH to wet the hair down.
3. Using a sharp pair of large scissors cut transversely immediately caudal to the occiput. Discard the body appropriately. *If removal of the head from the body is desired, follow this step. If not, go to step 4.*
4. Incise the cutaneous layers using the small scissors in a midline sagittal cut extending from caudal occiput to the rostrum, see Fig 8A. Displace the skin tissue laterally to visualize the skull.
5. Place the tips of the small scissors in the orbitals and cut the skull transversely as seen in Fig 8B.
6. Fixing the tips of the small scissors under the occiput of the skull, with slow rostral progression, cut on midline with small precise movements to the previous orbit incision, see Fig 8C.
7. Placing the forceps under the skull at the incision on the left side, grip and pull the skull laterally, perpendicular to the midline, peeling the skull from the brain, **see** Fig 8D.
8. If the skull breaks into pieces, remove each piece individually until complete hemisphere exposure is achieved.
9. Repeat steps 7 and 8 on the right hemisphere.
10. Once full brain exposure is accomplished, place the forceps beneath the brainstem and excise the brain by gently pulling superiorly and rostrally. *Inverting the mouse so that the dorsal surface of the cranium is closest to the workbench can be helpful, as it allows gravity to assist in brain extraction*.

**Fig 8:**
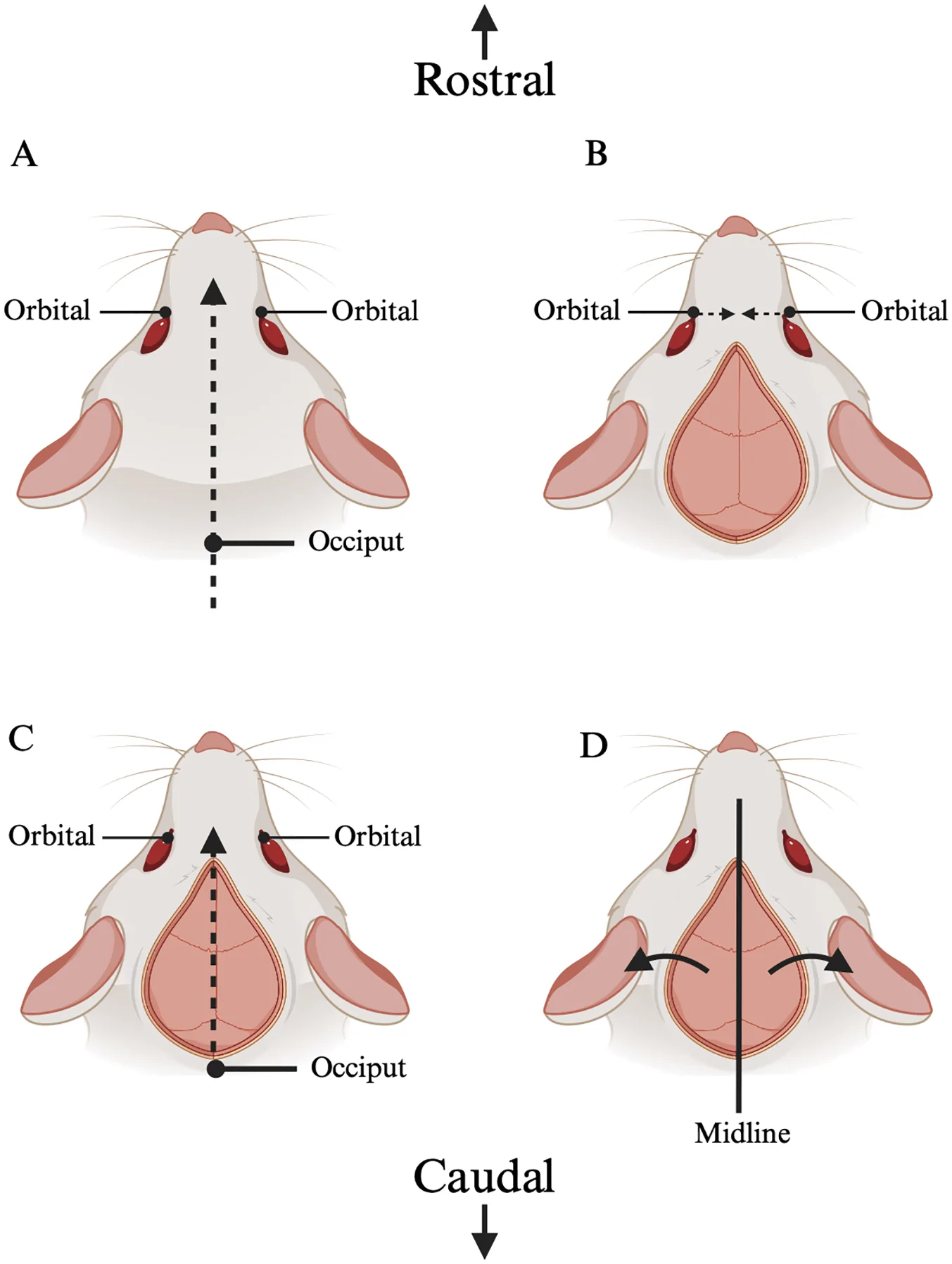
Brain Extraction. [A] Midline sagittal skin incision from occiput to rostrum. [B] Transverse incision between the orbitals. [C] Skull cut from occiput to orbit in a rostral direction. [D] Lateral separation of skull from brain. Created in BioRender. https://BioRender.com/k5txf1t.

### Brain microdissection

The following protocol described in this peer-reviewed article is published on protocols.io (https://dx.doi.org/10.17504/protocols.io.36wgqxq1ylk5/v2) and is included for printing purposes as S3 File.

The following procedure was performed under a microdissection microscope. Full dissection shown in Fig 9**.** See Fig 10 for a size reference of the mouse brain hemisphere after separation.

**Fig 9:**
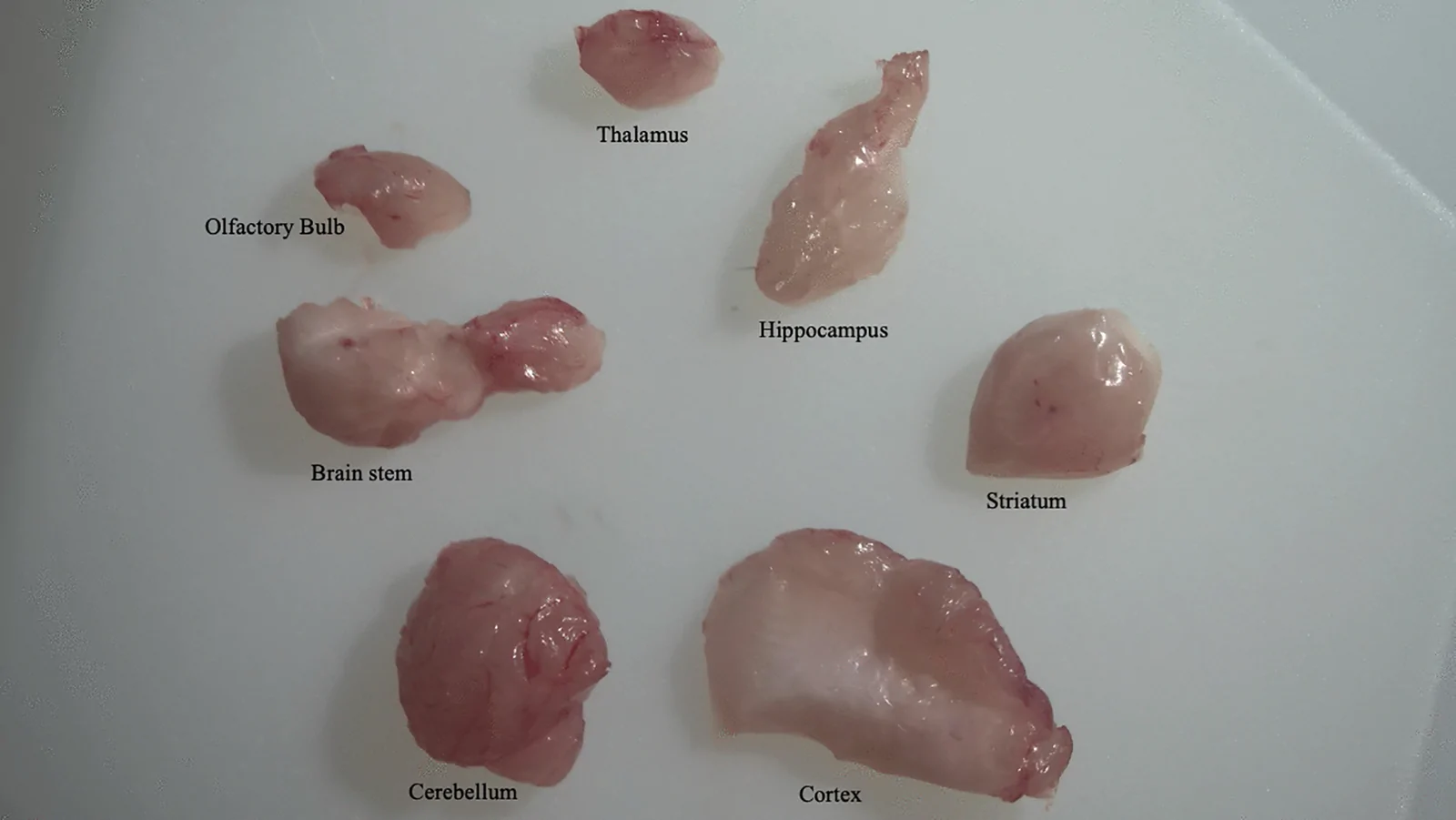
Completed Microdissection of the Mouse Brain. Labeled brain regions following careful dissection including the olfactory bulb, thalamus, hippocampus, striatum, brainstem, cerebellum, and cortex.

**Fig 10:**
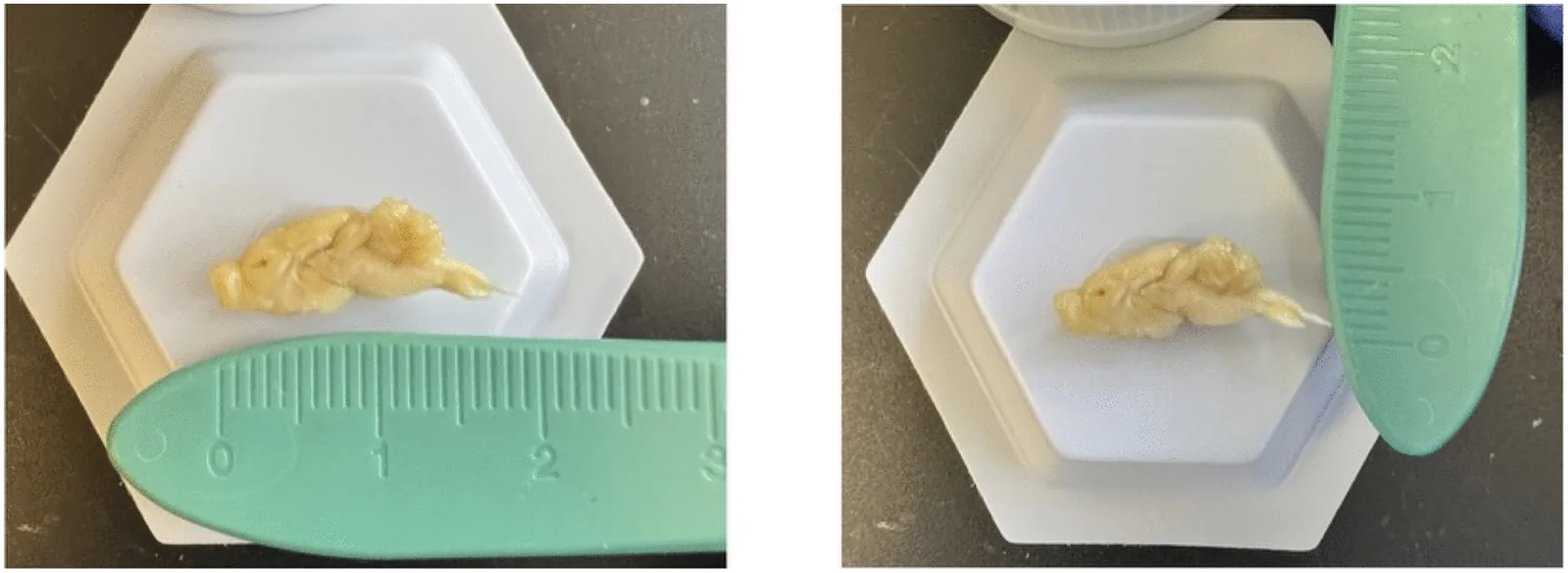
Size Reference of the Mouse Brain. Lateral-medial view of a fixed right hemisphere mouse brain. [A] Measuring approximately ~1.9 cm in length and **[B)**]~0.6 cm in height.

1. Hemispheric Separation—Place the brain on a clean, preferably chilled, surface with the dorsal surface facing upward and the ventral surface facing downward (Fig 11A). Using a scalpel, make a sagittal cut along the longitudinal fissure to separate the cerebral hemispheres (Fig 11B). For this step and the subsequent dissections, a chilled work surface may be prepared by placing wet ice and one to two small pieces of dry ice in a lidded Petri dish.
2. *Thalamus*—On the medial side of the brain while holding the hemisphere steady with forceps, visualize and scoop out the thalamus with the scalpel using blunt dissection paying attention to region boundaries, see Fig 11C.
3. *Hippocampus*— On the medial side of the brain, peel the cortex laterally, perpendicular to the midline using blunt dissection with the scalpel and while holding the hippocampus in place with the forceps to ensure separation of the two regions. Perform blunt dissection to extract the hippocampus medially toward the midline using the scalpel, see Fig 11D and E.
4. *Striatum*—Expose the striatum by removing the cortex and hippocampus as described above. Incise around the perimeter of the striatum with fine tip thumb forceps. Using the scalpel scoop out the striatum with reference to natural tissue separation, see Fig 11F. Striations help to differentiate the striatum from the cortex, see Fig 11D.
5. *Cortex*—Excise the base of the already dissected cortex using the scalpel blade, see Fig 11G.
6. *Cerebellum*—Remove the base of the cerebellum from the rest of the brain using a scalpel with attention to natural separation, see Fig 11H.
7. *Brainstem*—Excise the brain stem from the midbrain, see Fig 11I.
8. *Olfactory bulb*—Excise the olfactory bulb from the forebrain.

**Fig 11:**
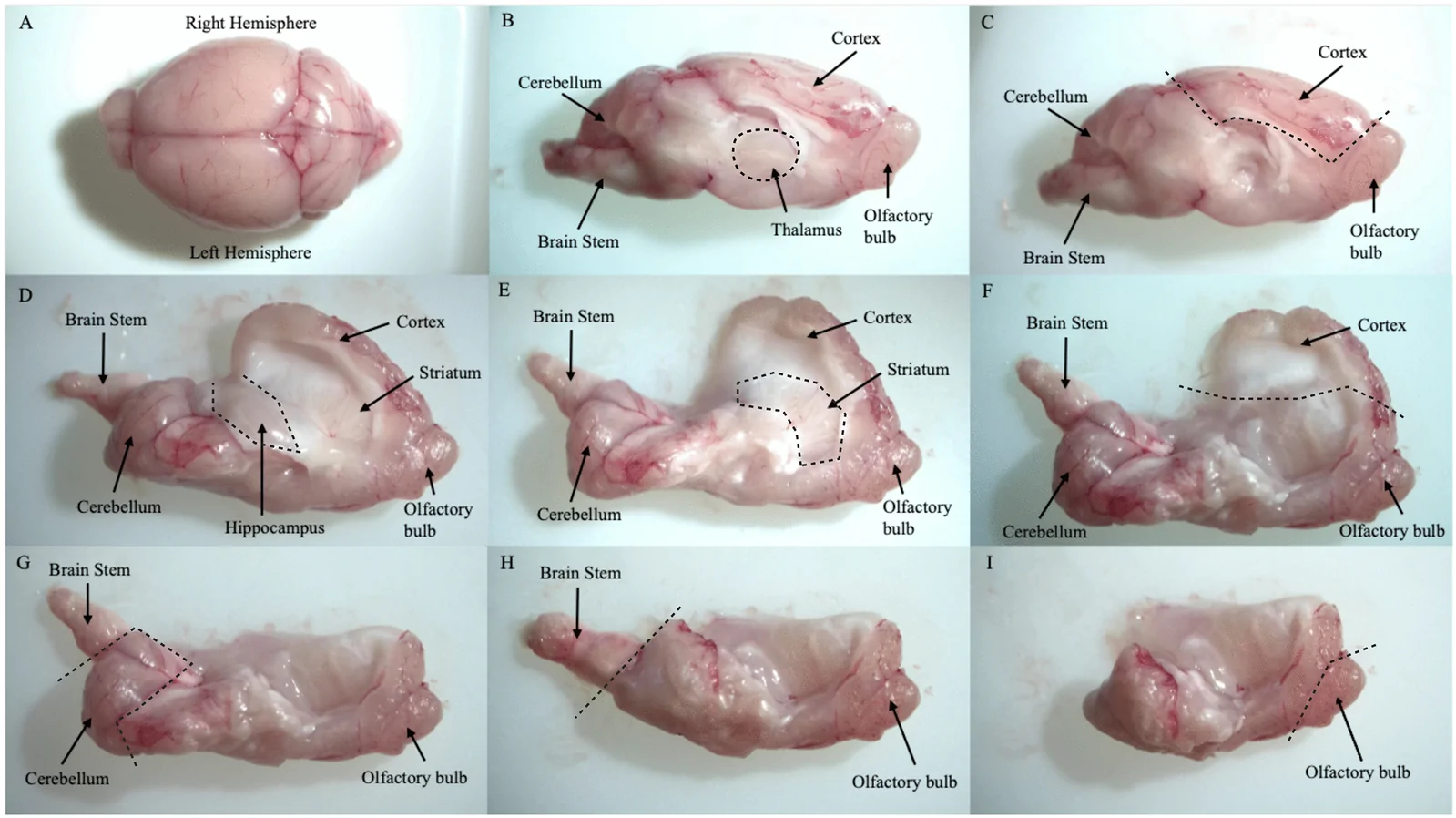
Brain Microdissection. Panel **[A]** shows the whole brain with labeled hemispheres. **[B]** sagittal view of left hemisphere **[C]** hemisphere without thalamus **[D]** cortex peeled back to reveal hippocampus and striatum **[E]** hemisphere without hippocampus, **[F]** hemisphere without striatum, **[G]** hemisphere without cortex, **[H]** hemisphere without cerebellum, [**I]** hemisphere without brain stem (Zeiss Stemi 508 & Axiocam 208 Color Photo). Created in BioRender. https://BioRender.com/8ei5ys0.

### Sciatic nerve

The following protocol described in this peer-reviewed article is published on protocols.io (https://dx.doi.org/10.17504/protocols.io.5qpvoed89l4o/v2) and is included for printing purposes as S4 File.

1. Place the mouse with the desired side up and pin the feet to the display tray.
2. Remove the skin over the quadriceps exposing the lateral surface.
3. Visualize the sciatic nerve and do not mistake the femur or muscle fascia for the sciatic nerve, see Fig 12.
4. Open surrounding muscle layers, following natural tissue separation, see Fig 12.
5. Once the sciatic nerve is exposed, leave the nerve in situ and fill the surrounding space with approximately 0.5 mL of 10% formalin using a 3 mL syringe. If fixation is desired, allow the tissue to fix for 30 minutes. Avoid stretching, manipulating, or otherwise disturbing the nerve, as sciatic nerve tissue is extremely delicate and easily damaged.
6. After the nerve has been fixed, cut the nerve at the head of femur down to the patella region.
7. Remove the nerve and preserve as desired.

**Fig 12:**
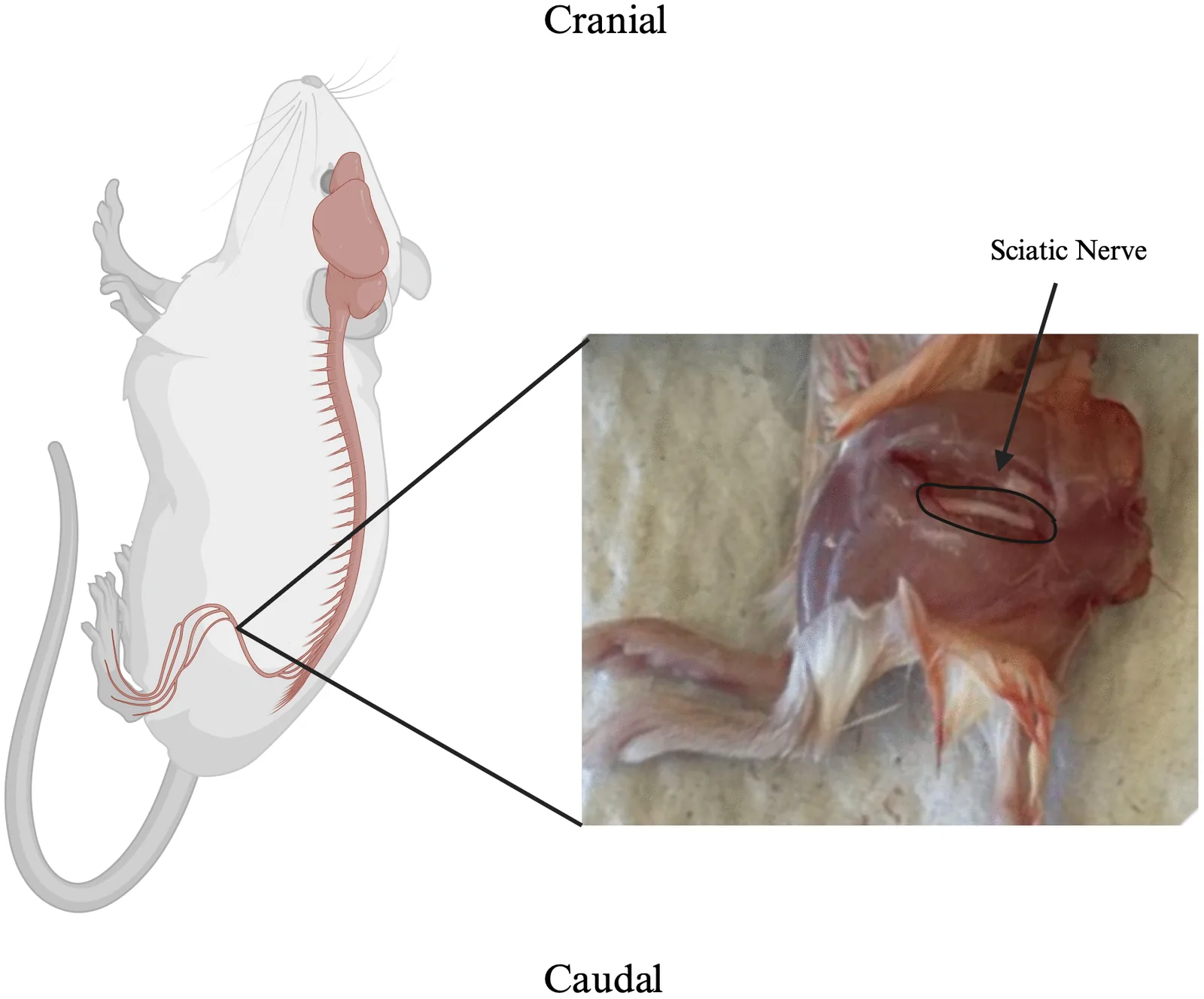
Proper positioning of an Anesthetized Mouse for Sciatic Nerve Extraction. The mouse is placed on its side with its hind limbs extended and secured to expose the sciatic nerve pathway, ensuring accurate and efficient nerve dissection. Created in BioRender. (2026) https://BioRender.com/yurd1hp.

### Spinal cord extrusion

The following protocol described in this peer-reviewed article is published on protocols.io (https://dx.doi.org/10.17504/protocols.io.dm6gp7181gzp/v2) and is included for printing purposes as S5 File.

1. Place mouse on ventrum and coat with EtOH.
2. Decapitate the mouse at the occiput.
3. Remove the skin covering the spine.
4. Perform a transverse scissor slice immediately cranial to the pelvis. This should extract the spine from the cervical vertebrae to the lumbosacral junction (Fig 13A)
5. While holding the body, lift the cut end on the spinal cord so there is enough space to fit syringe tip to the spinal cord cavity. Ensure the spinal cord is visible and remove excess tissue if necessary (Fig 13A).
6. Using a 10 ml syringe filled with chilled (~4°C) phosphate buffered saline (PBS), secure a tight seal with the syringe (may need to add a pipette tip with the tip cut to fit the size of the spinal cord) to the spinal cord cavity, apply firm force to plunger allowing hydrostatic pressure to extrude the spinal cord into a petri dish filled with phosphate buffered saline (PBS), see Fig 13D.
7. The extrusion of spinal cord may be easier if specific spinal regions are dissected. To do this, start at the cranial side of the body using the large scissors, slice through the tissues and ribs lateral to the spinal vertebrae progressing caudally to the pelvis (Fig 13B). If not already done, perform a transverse scissor slice immediately cranial to the pelvis (Fig 13C). Remove additional organs with forceps and scissors on the ventral side of the spine. Next, complete a transverse scissor slice at the thoraco-lumbar junction (cut just below last rib) to isolate the thoracic spinal cord from the lumbar spinal cord (Fig 13C). Hold the spinal cord in a fashion that eliminates the curvature and proceed with extrusion. If the spinal cord is stuck to the end of the vertebrae, carefully use the forceps to manipulate the spinal cord out of the cavity.
8. Store as desired.

**Fig 13:**
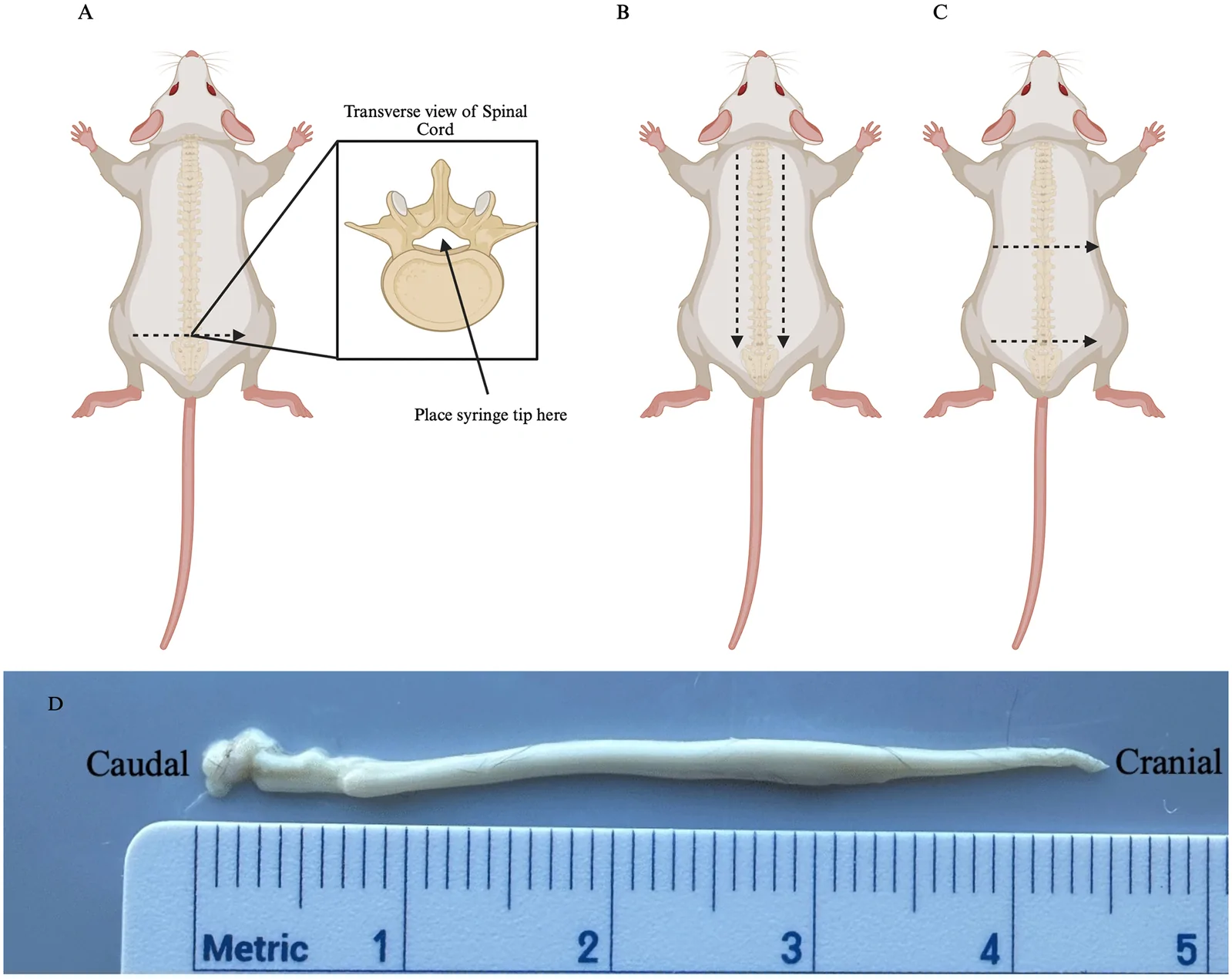
Spinal Cord Post-Extraction. [A] Transverse scissor slice cranial to the pelvis with a transverse view of the spinal cord; [B] Two slices lateral to the spinal vertebrae; [C] One slice at the thoraco-lumbar junction and one slice immediately cranial to the pelvis. [D] Whole spinal cord post-extraction. Created in BioRender. https://BioRender.com/4olnrjf.

## Expected results

It’s important to ensure that each procedure is performed efficiently with minimal damage to tissues and samples. See Table 2 for the recommended time to complete each protocol and criteria for successful samples.

**Table 2:** Recommended times to complete each protocol and criteria for successful samples.

Protocol	Recommended Time for Protocol	Criteria for Successful Samples
CSF Collection	CSF should be collected in a timely manner, approximately in 2–3 minutes as the mouse is under anesthesia.	4-10ul of uncontaminated CSF. No hemorrhage contamination is optimal. If the sample has hemorrhage contamination, it may be spun down and transferred to a new tube depending on sensitivity of assay. Sample acquisition must occur while the mouse is alive, after death CSF is extremely difficult to obtain.
Brain Extraction	1 to 2 minutes from time of decapitation is optimal for maintaining tissue integrity.	Hemispheres remain intact; very little to no damage from instruments.
Brain Microdissection	5 to 7 minutes, less time is more optimal to maximize preservation of critical proteins or desired experimental elements. Ice underneath the weigh boat may help keep the tissues cool for preservation and to maintain tissue integrity when dissecting regions.	Regions are properly identified, isolated, and dissected and preserved or frozen promptly to maintain desired experimental elements.
Sciatic Nerve Extraction	1 to 2 minutes plus 30 minutes for fixation.	Minimal damage to nerve. Intact, no pulling or stretching, with clean cuts at either end.
Spinal Cord Extrusion	1 to 5 minutes.	Spinal cord is intact or separated into specific spinal regions. Little to no damage by syringe or thumb forceps.

### CSF collection in neurodegenerative disease studies

CSF is the fluid surrounding the central nervous system (CNS), providing needed nutrients and removing waste products. Analysis of the CSF gives real-time insight into the biochemical state of the CNS, such as biomarker detection, disease progression tracking, and pharmaceutical testing [20]. In mice and most rodents, the cisterna magna is the typical site for CSF collection due to its easier accessibility and large volume [15]. Obtaining sufficient volume and minimizing hemorrhage contamination is critical when collecting CSF. Evidence of sample quantity, quality, and reproducibility using our technique is displayed in Fig 14 showing reproducible protein concentrations between samples in biohazardous prion diseased transgenic mouse models.

**Fig 14:**
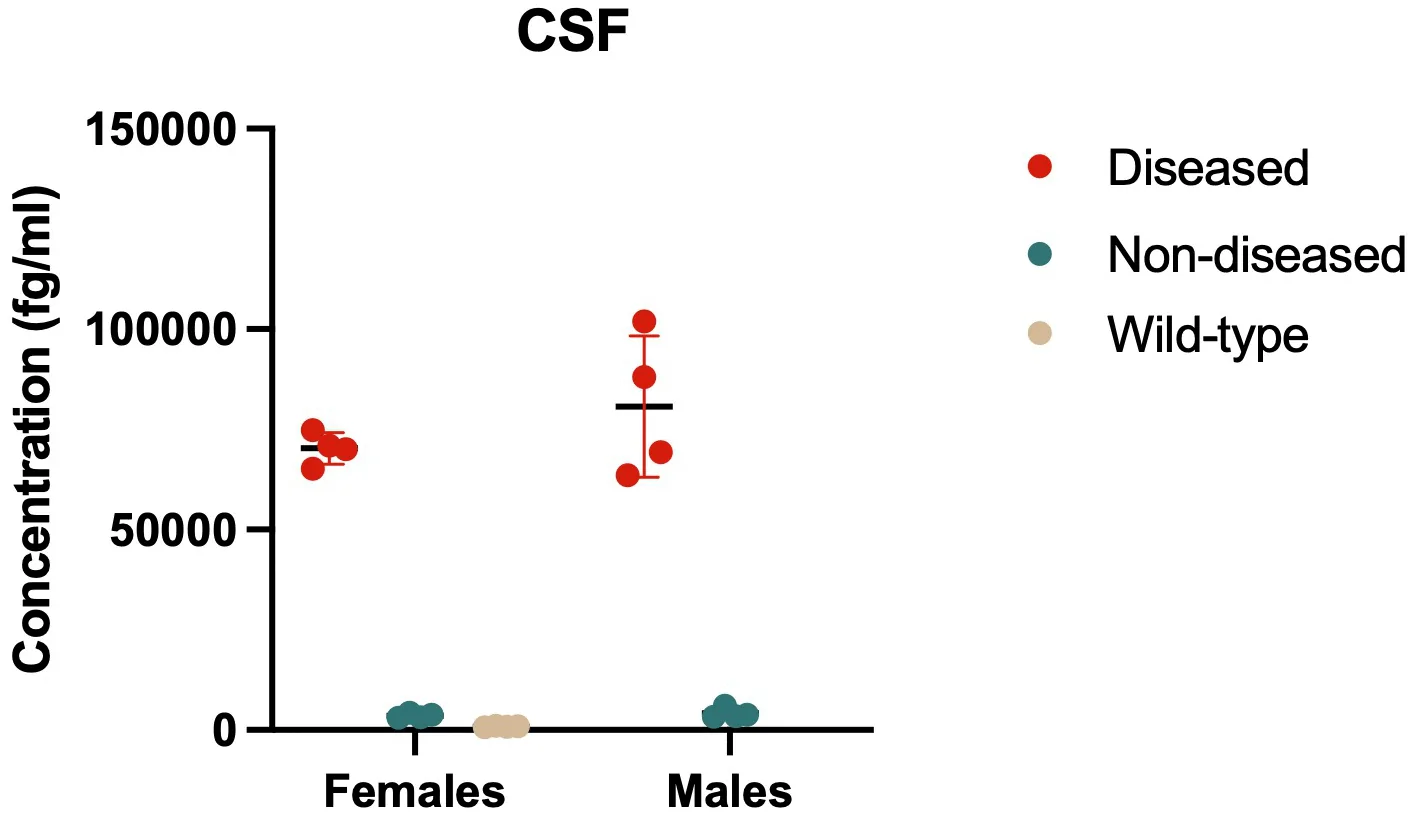
Total Tau protein concentration levels detected in CSF displaying reproducible scientific data. Prion diseased transgenic mice (red, n = 8), non-diseased transgenic mice (blue, n = 8), wild-type mice (tan, n = 4).

### Brain extraction in neurodegenerative disease studies

Whole-brain dissection is a foundational technique to acquire tissue for many methods in neurodegenerative disease studies. Among these methods, histology is a commonly used to analyze basic morphology and the quantity of specific cell types or proteins of interest, requiring precise extraction to maintain tissue integrity. Histologic examples of brain hemispheres are shown displaying spongiform change surrounding the dentate gyrus of the hippocampus in transgenic mice infected with a prion disease (Fig 15A) when compared to a control (Fig 15B).

**Fig 15:**
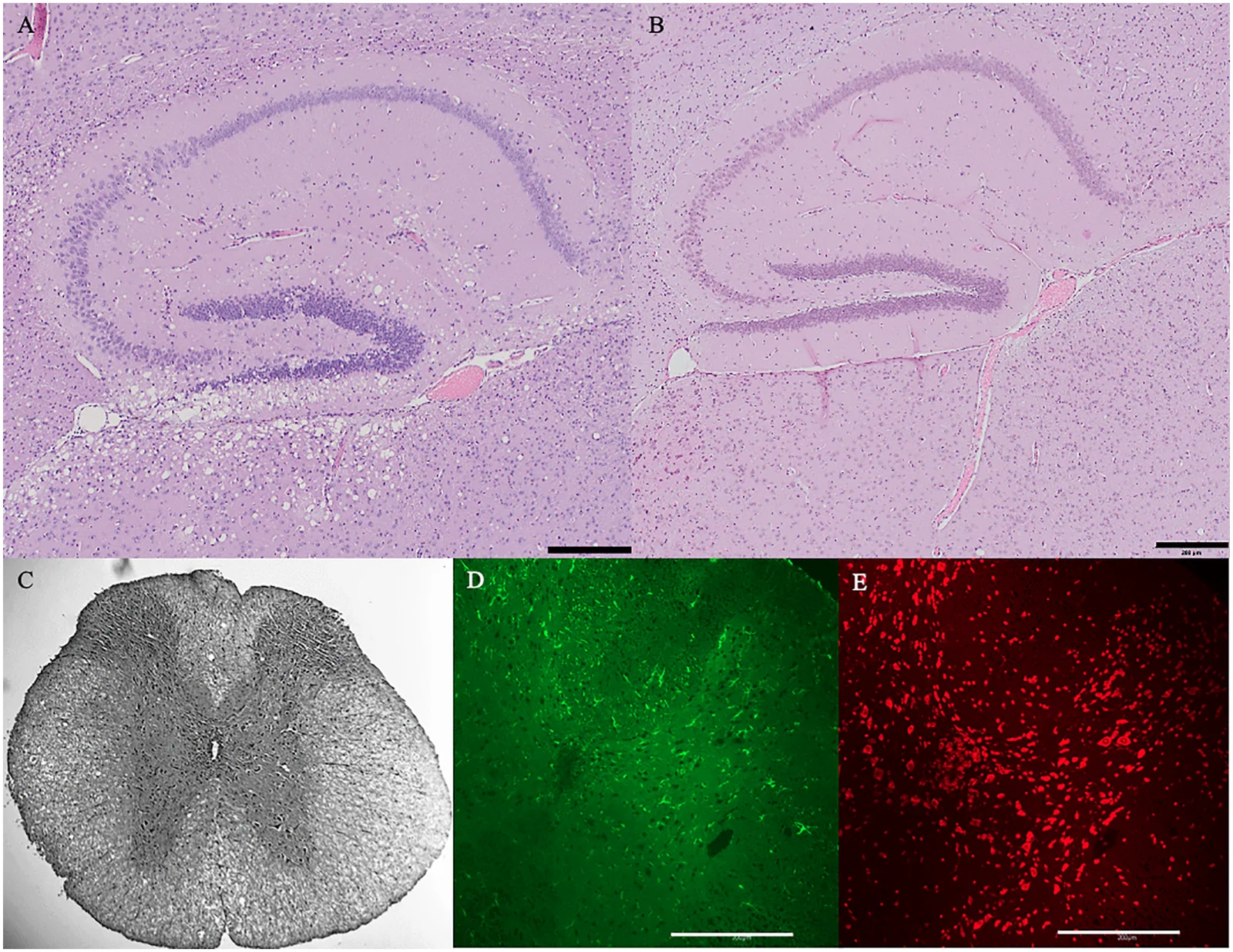
Examples of histologic preparations after brain extraction and spinal cord extrusion demonstrating the importance of maintaining tissue integrity for histologic studies. **[A]** Chronic wasting disease (CWD) transgenic mouse model brain hemisphere sections processed and stained with hematoxylin and eosin (H&E) exhibiting spongiform tissue around the dentate gyrus of the hippocampus as opposed to non-diseased control brain in panel **[B].** Scale bar 200um. **[C]** Spinal cord section processed and stained with H&E to display spinal cord morphology, shown in black and white. **[D]** Presence of astrocytes (green) in spinal cord sections after immunofluorescence (IF) staining in CWD mouse models, scale bar 200um. **[E]** Spinal cord section of IF neuronal labeling (red), scale bar 200um.

### Microdissected brain regions in neurodegenerative disease studies

Often, neurodegenerative disease studies require dissection of specific brain regions due to specific region neuropathology. For example, prion diseases cause morphological changes in the hippocampus and cortex regions of the brain [33,34], which require precise isolation for use in research. In relation to Huntington’s disease, studies are typically focused on the striatum as it is the main region affected [4,10]. Similarly, biomarker research in neurological studies is most accurate and reproducible when conducted on distinct sections of the brain because it eliminates data dilution from regions that may have less pathology. These methods are also important in pharmacology development studies as brain regions are often required to better understand pharmaceutical drug distribution, safety, and mechanisms of action [35].

### Sciatic nerve in neurodegenerative disease studies

The largest and longest nerve in the rodent and human body is the sciatic nerve. It extends from the pelvic region and tracks along the hind limbs. Due to its size and simple accessibility, this peripheral nerve is integral to nerve studies. As it is part of the peripheral nervous system (PNS), it can give information about peripheral neuropathies, neuromuscular junction processes, therapeutics, and Wallerian degeneration [16,22,36].

### Spinal cord in neurodegenerative disease studies

The dynamic conduit for connection between the brain and body of sensory and motor signaling is the latter half of the CNS, the spinal cord. Spinal cord research gives insight into motor neuron diseases, degeneration and regeneration processes, inflammation, and traumatic injuries. Extrusion of the spinal cord is commonly performed via hydrostatic pressure, which allows gentle expulsion without tissue disturbance or contamination [17]. An example of this is shown (Fig 15C) with hematoxylin and eosin staining of spinal cord sections using this extrusion protocol. Immunofluorescent staining displays neurons (in red) and astrogliosis (in green) in cervidized transgenic mice infected with chronic wasting disease (CWD), a fatal prion disease found in cervids (Fig 15D and E).

### Validation of methods in previous literature

The accuracy, quality, and reproducibility of obtaining tissue and CSF samples using our methods are further exemplified by several previous publications encompassing many different models of neurodegeneration producing valid scientific results. A brief description of how our methods were applied in research studies is included.

Striatum regions were dissected to characterize early transcriptomic, molecular, and cellular phenotypes in six distinct knock-in mouse models of the Huntington’s disease (HD) mutation in a time course sampling between 4 and 20 postnatal weeks. The emergence of a transcriptomic signature in the mice was discovered involving hundreds of differentially expressed genes and changes in diverse molecular pathways. Somatic CAG-length instability was also assessed from the micro dissected striatum sections. Whole brain hemispheres displayed the timing onset of mutant huntingtin nuclear localization phenotypes. This work provides a foundation for understanding the earliest transcriptional and molecular changes contributing to HD pathogenesis [4].

CSF, whole brain hemispheres, and cortex and striatum micro dissected brain regions were obtained to analyze the effects of knocking out the huntingtin (HTT) protein recapitulating current Huntington’s disease therapies. Three different lines of transgenic mouse model samples were analyzed to characterize the inducible HTT knock-out models (micro dissected cortex and striatum), confirm continued HTT reduction throughout the study (micro dissected cortex and striatum), display increased pathologic calcium accumulation in the thalamus (whole brain hemisphere), monitor brain biomarker levels (CSF and whole brain hemisphere), and determine the presence of neurons, astrocytes, and microglia (whole brain hemisphere) [10].

Whole brain hemispheres were dissected to characterize a transgenic mouse model of the human neurodevelopmental disorder Spastic Tetraplegia, Thin Corpus Callosum, and Progressive Microcephaly (SPATCCM) in which coronal sections of cortex layer 1 and corpus callosum were measured and compared to wild type, showing significant size differences. Histologic assessments were performed to determine presence of neurons and distribution of myelin basic protein [11].

Whole brain hemispheres, CSF, and sciatic nerve samples were utilized in a study reporting prion protein (PrP) turnover in vivo and the time to effect of prion disease therapeutics. Targeted mass spectrometry and antisense oligonucleotide treatment followed by timed PrP measurement was used to estimate a half-life of 5–6 days for PrP in the brain which will inform the design of both preclinical and clinical studies of PrP-lowering drugs [37].

Biohazardous whole brain hemispheres were dissected in the biosafety cabinet to analyze the morphologic impacts of formic acid used commonly for prion decontamination. Cortex and hippocampus measurements in the sagittal plane were performed along with histologic assessments requiring high quality, reproducible samples of whole brain dissections [38].

### Dissection troubleshooting

It may take some time for inexperienced personnel to refine their skills; however, there are many errors that can occur during dissections, and it’s important to recognize potential mistakes and avoid them when possible. It is crucial to treat all tissues in dissections with care by allowing ample time to meticulously extract them. The quality of samples can be severely impacted by rushing through the protocol and not being mindful of how the instruments are used or how the tissues are handled. In most cases, such as in the brain, sciatic nerve, and spinal cord extrusion, it’s imperative that tissues are completely exposed before extraction. This will help to limit unnecessary damage. Consulting other resources like the Allen Brain Atlas will also help personnel to build background knowledge and get familiar with the anatomy and regions that these protocols focus on. Specific troubleshooting based on protocol is addressed in Table 3.

**Table 3:** Common errors and troubleshooting solutions arranged by protocol.

Protocol	Common Errors	Solutions
Brain Extraction	Poor skull exposure	Confirm full exposure by viewing all sides of the mouse brain before trying to remove it from the body.
	Brain damaged during extraction	Confirm proper instruments and take extra precautions to avoid unnecessary handling of brain.
	Dissection at warm room temperature leading to poor quality specimens	Use ice or a cool surface underneath the brain. Transfer specimen to ice when no longer in use. Be careful to not freeze tissue during dissection.
Brain Microdissection	Contaminated specimens	Review anatomy and use sharp tools to ensure quality extractions. Pay special attention to natural tissue separation.
	Extreme manipulations of specimens	Start with minimal contact of tissues to feel for tissues changes.
	Failure to isolate regions	Utilize a dissection microscope or other instrument that would improve field of view.
	Dissection at warm room temperature leading to poor quality specimens	Use ice or a cool surface underneath the brain. Transfer specimen to ice when no longer in use. Be careful to not freeze tissue during dissection.
CSF Collection	Blood in capillary tube	Review the anatomical structure of cisterna magna and blot region with sterile swab. Avoid puncturing underlying vasculature beneath the arachnoid membrane.
Sciatic Nerve Dissection	Mistaking femur for sciatic nerve	Apply a slight tug on the visualized nerve, if the nerve does not give, try and relocate to the nerve.
	Damage to sciatic nerve	Ensure the entire nerve is covered by fixative for an appropriate time before removal from leg.
Spinal Cord Extrusion	Failure to extrude spinal cord with hydrostatic pressure	Ensure visualization of spinal cord at both ends prior to hydrostatic expulsion. If the ends are not visualized, take small cuts from non-visualized end(s) and repeat. If error persists, ensure tight seal of syringe to spinal cord cavity.

## Discussion

High-quality, reproducible dissection protocol techniques are the backbone of experimental neuroscience. Whether the focus is on the CNS or PNS, consistency ensures that biological variability drives the observed outcomes, not technical error [6]. These protocols maintain a high level of tissue integrity that allows for cross-laboratory collaborative comparison, which is a necessity in translational research [7,17]. Optimization also reduces animal usage, maximizes data per specimen, and minimizes overall suffering. As neuroscience research continues to intersect with technology, clinical medicine, and ethics, standardized protocols are called to transcend previous frameworks/compositions and establish new benchmarks for precision, reproducibility, and ethical rigor [5].

These protocols lay the groundwork for translational advances and bridge laboratory practices to clinical skills. Precise anatomical knowledge directly translates into surgical practices, where understanding the location and relationships of structures guides every move. Dissections like these may even be useful for training future physicians by developing fine motor technical skills with refined surgical tools, which are required in microsurgery settings [39]. Performing brain microdissections under the microscope also builds a better understanding of how to work with varied depth-of-field, similar to robotic surgery skills that occur under magnification, enhancing operative medicine. Lastly, adherence to biosafety protocols (BSL-2) during dissections mirrors the sterile techniques of surgical suites, not only reinforcing good habits but once again preventing contamination. These practices protect both the research and tissue integrity to model the necessary standards for plausible and reputable research findings.

## Conclusion

In closing, implementation of standardized protocols across the brain, spinal cord, CSF, and peripheral nerves such as these provided, allows for maximized tissue specimen value, reduced animal usage, and more robust experimental power. Furthermore, it encourages cross disciplinary benefits in pharmaceutical, forensic, bioengineering, and regenerative medicine, allowing for a broader impact.

## Supporting information

S1 File Step-by-step protocol, also available on protocols.io. Protocol describes method for mouse CSF collection.
(PDF)

S2 File Step-by-step protocol, also available on protocols.io. Protocol describes method for mouse brain collection.
(PDF)

S3 File Step-by-step protocol, also available on protocols.io. Protocol describes method for mouse brain microdissection.
(PDF)

S4 File Step-by-step protocol, also available on protocols.io. Protocol describes method for mouse sciatic nerve collection.
(PDF)

S5 File Step-by-step protocol, also available on protocols.io. Protocol describes method for mouse spinal cord collection via hydropulsion technique.
(PDF)
