# Supplementary material for: Neurological tissue dissection techniques in mouse models for reproducible scientific results: Brain, spinal cord, cerebrospinal fluid, and sciatic nerve
Source: PLoS One. 2026 Oct 7;21(10):e0358771. doi: 10.1371/journal.pone.0358771 (PMC13645030; doi:10.1371/journal.pone.0358771)
Supplement: Supplementary file 1 [file pone.0358771.s001.pdf]

Aug 06, 2026 Version 2

# Cerebrospinal Fluid (CSF) Collection in mouse models V.2

DOI
https://dx.doi.org/10.17504/protocols.io.36wgqpnwxvk5/v2

Lilia Crew[1], Alyssa Seerley[2], Serena McElroy[2], Andrea Grindeland Panter[1,2], Lilia Crew[1,2]

[1]Touro University College of Osteopathic Medicine, Great Falls, MT, United States;
[2]Weissman Hood Institute at Touro University, McLaughlin Research Institute, Great Falls, MT, United States

Lilia Crew: Co-first author;
Alyssa Seerley: Co-first author
Andrea Grindeland Panter: Senior author

Alyssa Seerley
Weissman Hood Institute at Touro University; McLaughlin Rese...

## Create & collaborate more with a free account

Edit and publish protocols, collaborate in communities, share insights through comments, and track progress with run records.

Create free account

OPEN ACCESS

DOI: https://dx.doi.org/10.17504/protocols.io.36wgqpnwxvk5/v2

**Protocol Citation:**
Lilia Crew, Alyssa Seerley, Serena McElroy, Andrea Grindeland Panter, Lilia Crew 2026. Cerebrospinal Fluid (CSF) Collection in mouse models . **protocols.io**
https://dx.doi.org/10.17504/protocols.io.36wgqpnwxvk5/v2
Version created by
Alyssa Seerley

**License:** This is an open access protocol distributed under the terms of the **Creative Commons Attribution License**, which permits unrestricted use, distribution, and reproduction in any medium, provided the original author and source are credited

**Protocol status:** Working
**We use this protocol and it's working**

**Created:** August 06, 2026

**Last Modified:** August 06, 2026

**Protocol Integer ID:** 322080

**Keywords:** Cerebrospinal fluid, CSF, mouse models, Neurodegeneration, cerebrospinal fluid, csf, mouse model, collection in mouse model, mouse

**Funders Acknowledgements:**
**NIH General Medical Sciences**
**Grant ID: 5P20GM152335**

## Abstract

This protocol describes a method for the collection of Cerebrospinal fluid (CSF) from a mouse able to be used for downstream analysis.

## Materials

Large Scissors
Small Scissors
70% EtOH
Fine Forceps (Dumont Curved)
Capillary tube made of polished borosilicate glass with filament, size O.D.:1.0mm, I.D.: 0.78mm.
Dissection microscope

## Terminal Cerebrospinal Fluid (CSF) Collection

1 Apply 70% ethanol (EtOH) to the surgical area to wet down the hair of the mouse.

2 Place the mouse in prone or sternal recumbency position. Using a #10 scalpel blade, create a midline skin incision extending from the occiput to the level of the second cervical vertebra (C2). If needed, gently flex and extend the skull to identify the precise location of the occiput before making the incision.

3 Maintain the mouse in sternal recumbency with the skull in flexion. Using sharp dissection, incise along the midline between the cervical musculature from the occiput to C2. The cervical muscles rhomboideus cervicis, the cervical portion of the trapezius, splenius capitis, semispinalis capitis, and erector spinae muscles should be separated at the midline to expose the cisterna magna. Deeper muscles that are difficult to visualize grossly may be separated under the dissecting microscope in Step 6 using blunt dissection techniques.

4 Place the ventral surface of the thorax of the mouse on the weight boat under the microscope.

5 Stabilize the mouse, ensuring that the skull is hyper flexed for maximum access to the cisterna magna. See Fig. 1

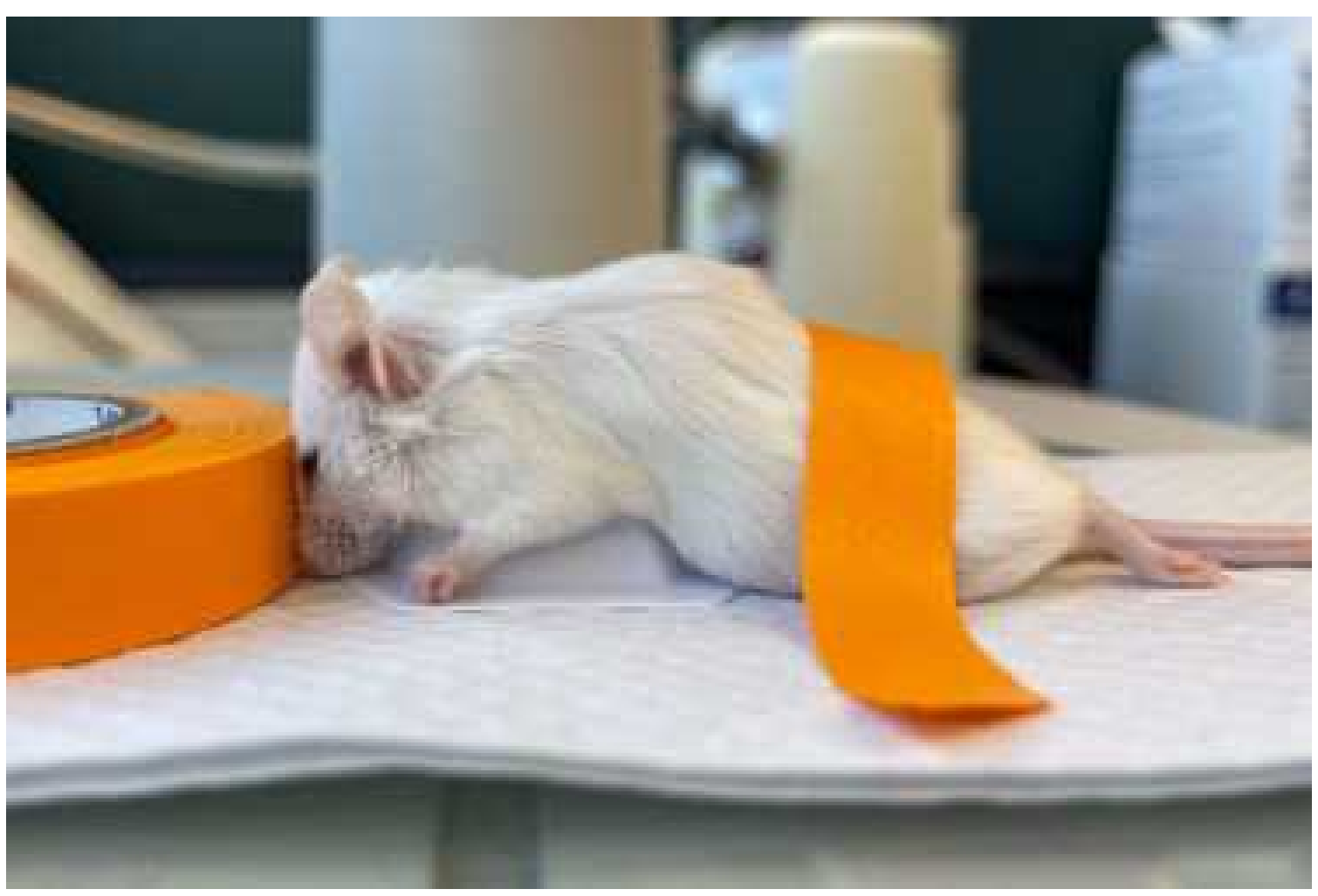

**Fig. 1 Mouse Positioning to obtain CSF.** Anesthetized mouse positioned for cerebrospinal fluid (CSF) collection, demonstrating hyperflexion in the cranial-occipital region. The mouse is secured with orange tape to maintain the necessary posture for effective CSF extraction and hyperflexion is induced via a weigh boat and tape roll or any object that will hold the correct stabilization and placement of the head.

6 Bluntly dissect and separate at midline the interior muscles remaining from step 3 down to the cisterna magna to visualize a transparent membrane. This is the arachnoid membrane covering the cisterna magna, which is a large pocket of CSF within the subarachnoid space. If hemorrhage from the musculature occurs, blot it with a cotton swab prior to puncturing the arachnoid membrane as it is critical to avoid blood contamination in the CSF, see Fig.2.

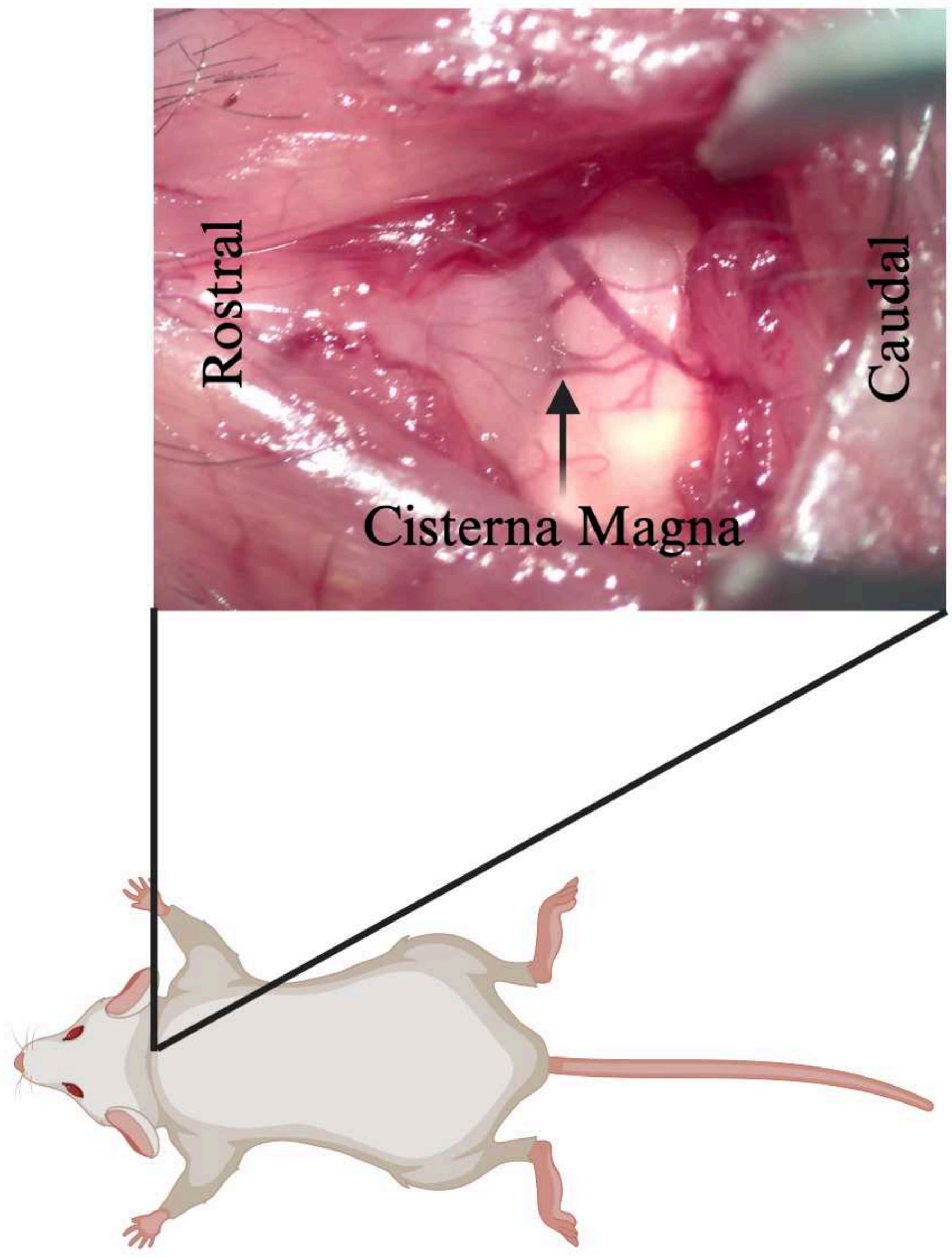


**Fig 2. Visualization of cisterna magna.** Created in BioRender.

7 Once the arachnoid membrane is visualized, use small thumb forceps to move the muscles laterally if needed for complete visualization.

8 With the capillary tube, puncture the arachnoid membrane at a 45° angle, using a gentle spinning motion.

Note

Using a micropipette puller, make a needle from a 10 cm length capillary tube made of polished borosilicate glass with filament, size O.D.:1.0mm I.D.: 0.78mm. The end of the needle will need to be cut to a diameter of 0.2mm, as the larger diameter will allow the CSF to flow into the tube by capillary action. See Fig 2.

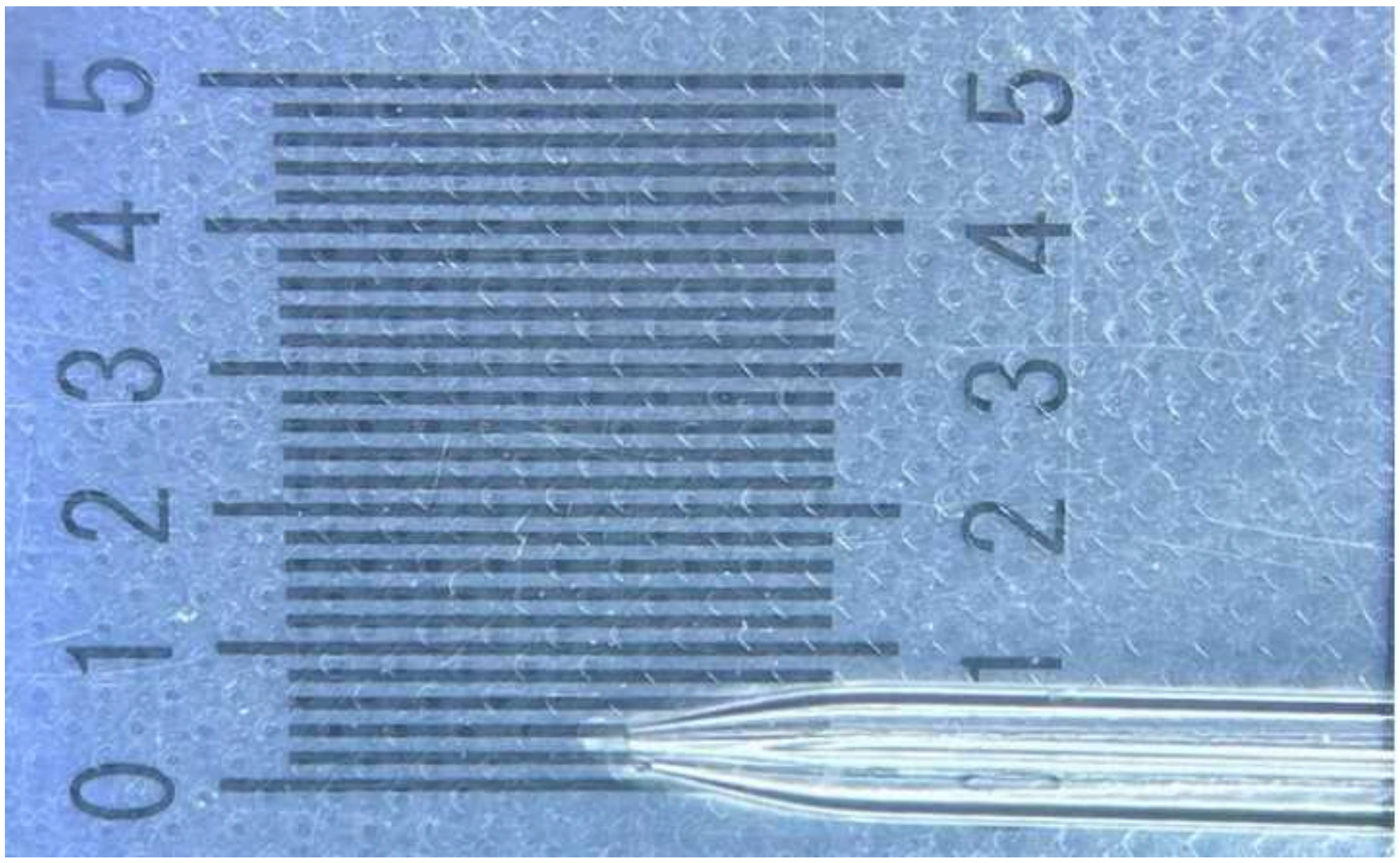


**Fig.3 Capillary tube recommendations.** Borosilicate glass with filament [O.D.: 1.0mm, I.D.: 0.78mm, 10cm length] capillary tube pulled into a needle and cut leaving a 0.2mm diameter opening for puncture of the arachidonic membrane.

9 Allow CSF to flow into the tube by capillary action. The approximate amount of CSF collected is between 4-10µL. See Fig 4.

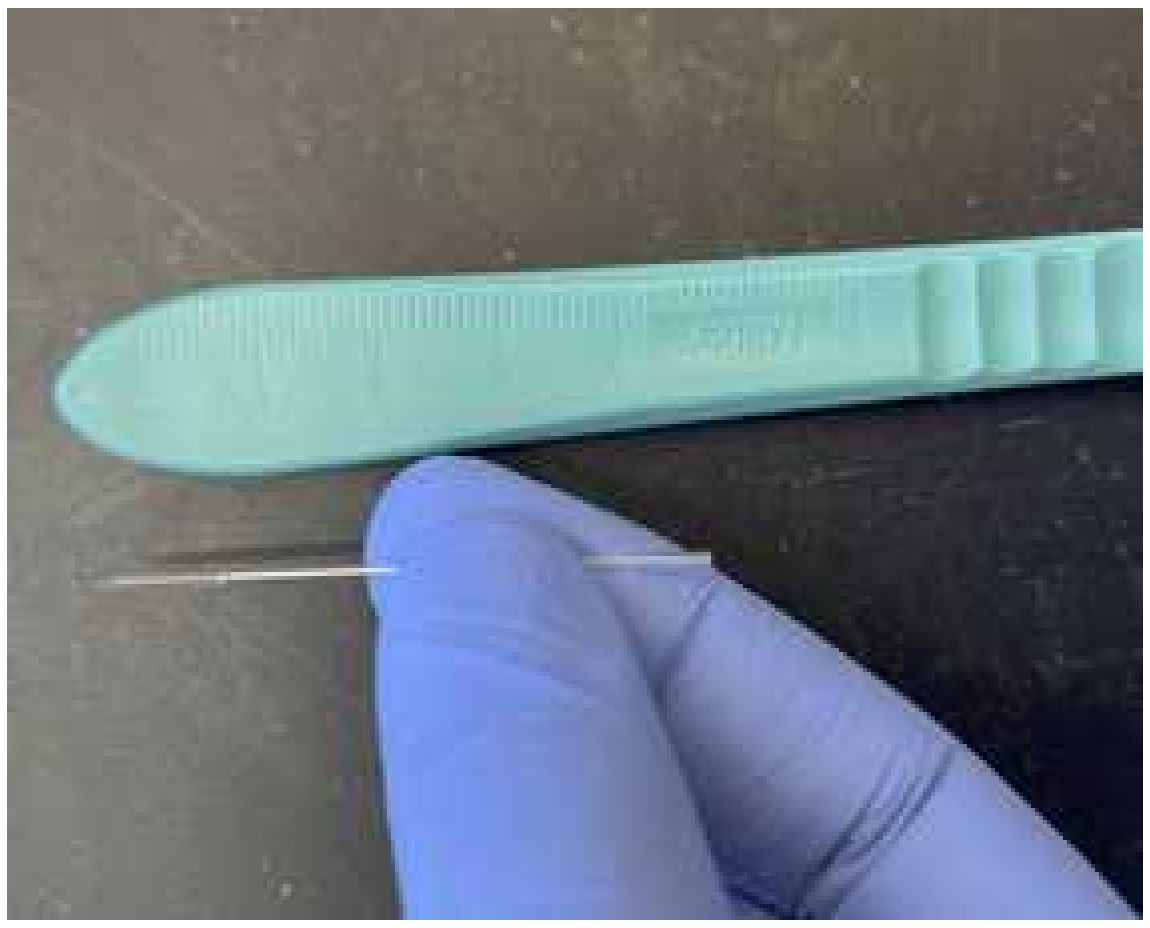

**Fig. 4 Capillary tube containing CSF.**

10 Place the blunt end of the capillary tube into the rubber bulb capillary dispenser instrument and expel the sample into the desired sample tube. See Fig. 4

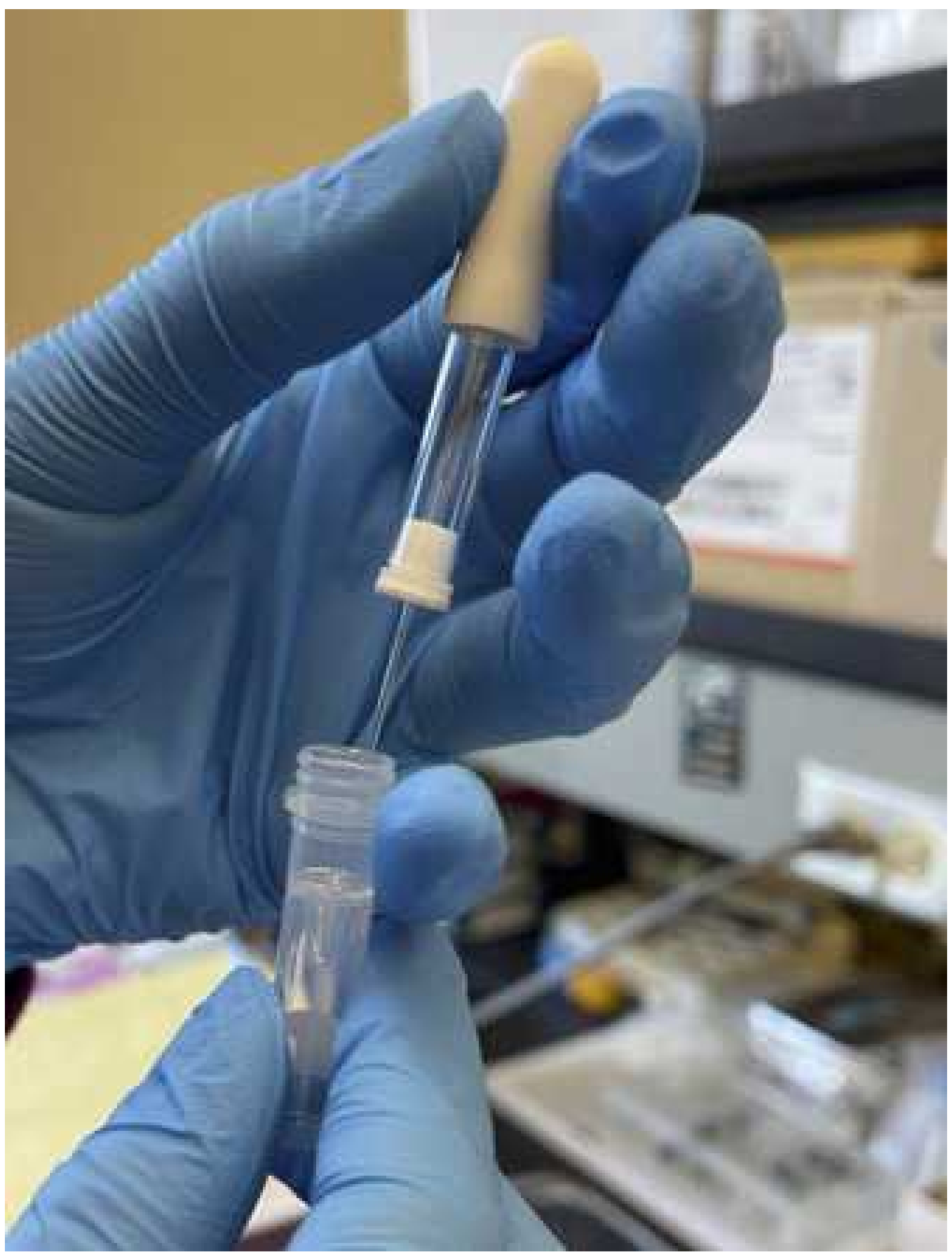

**Fig. 5 Transference of Collected CSF.** Using a rubber bulb capillary dispenser into a storage vial in a laboratory setting. This process ensures the integrity and sterility of the sample for subsequent analyses.

## Acknowledgements

Colony management for the mouse models included in this study was provided by the McLaughlin Research Institute - Gene Editing and Mouse Models Assessment (GEMMA) Core Facility within the Center for Integrated Biomedical and Rural Health Research, RRID: SCR_027045, 1P20GM152335. The authors thank Rose Pitstick and Kaela Davey for exceptional care of the mice. We would also like to thank student intern research assistant Bridget Gray and federal work study Touro COM-MT students Dalia Shaaban and Clairissa Kaylor for thoughtful discussions regarding planning and presentation of dissection methods in the mouse brain microdissections.
