# Supplementary material for: Neurological tissue dissection techniques in mouse models for reproducible scientific results: Brain, spinal cord, cerebrospinal fluid, and sciatic nerve
Source: PLoS One. 2026 Oct 7;21(10):e0358771. doi: 10.1371/journal.pone.0358771 (PMC13645030; doi:10.1371/journal.pone.0358771)
Supplement: Supplementary file 2 [file pone.0358771.s002.pdf]

Aug 06, 2026 Version 2

Mouse Brain Extraction V.2

DOI
https://dx.doi.org/10.17504/protocols.io.6qpvrbqy3lmk/v2

Lilia Crew[1], Alyssa Seerley[2], Serena McElroy[2], Andrea Grindeland Panter[1,2], Lilia Crew[1,2]

[1]Touro University College of Osteopathic Medicine, Great Falls, MT, United States;
[2]Weissman Hood Institute at Touro University, McLaughlin Research Institute, Great Falls, MT, United States

Lilia Crew: Co-first author;
Alyssa Seerley: Co-first author
Andrea Grindeland Panter: Senior author

Alyssa Seerley
Weissman Hood Institute at Touro University; McLaughlin Rese...

Create & collaborate more with a free account

Edit and publish protocols, collaborate in communities, share insights through comments, and track progress with run records.

Create free account

OPEN ACCESS

DOI: https://dx.doi.org/10.17504/protocols.io.6qpvrbqy3lmk/v2

Protocol Citation:
Lilia Crew, Alyssa Seerley, Serena McElroy, Andrea Grindeland Panter, Lilia Crew 2026. Mouse Brain Extraction. **protocols.io**
https://dx.doi.org/10.17504/protocols.io.6qpvrbqy3lmk/v2
Version created by
Alyssa Seerley

License: This is an open access protocol distributed under the terms of the **Creative Commons Attribution License**, which permits unrestricted use, distribution, and reproduction in any medium, provided the original author and source are credited

Protocol status: Working
We use this protocol and it's working

Created: August 06, 2026

Last Modified: August 06, 2026

Protocol Integer ID: 322107

Keywords: Mouse, brain extraction, mouse brain extraction this protocol, mouse brain extraction, intact mouse brain, method for the extraction, extraction, downstream analysis, mouse

Funders Acknowledgements:
NIH General Medical Sciences
Grant ID: P20GM152335

Abstract

This protocol describes a method for the extraction of an intact mouse brain to be used for downstream analysis.

Materials

70% EtOH
Large scissors
Small scissors
Forceps

Mouse Brain Extraction

1 Place the mouse in a ventral position

2 Spray the cranium with 70% EtOH to wet the hair down

3 Using a sharp pair of large scissors cut transversely immediately caudal to the occiput. Discard the body appropriately. *If removal of the head from the body is desired, follow this step. If not, go to step 4.*

4 Incise the cutaneous layers using the small scissors in a midline sagittal cut extending from caudal occiput to the rostrum, see **Fig 1A**. Displace the skin tissue laterally to visualize the skull.

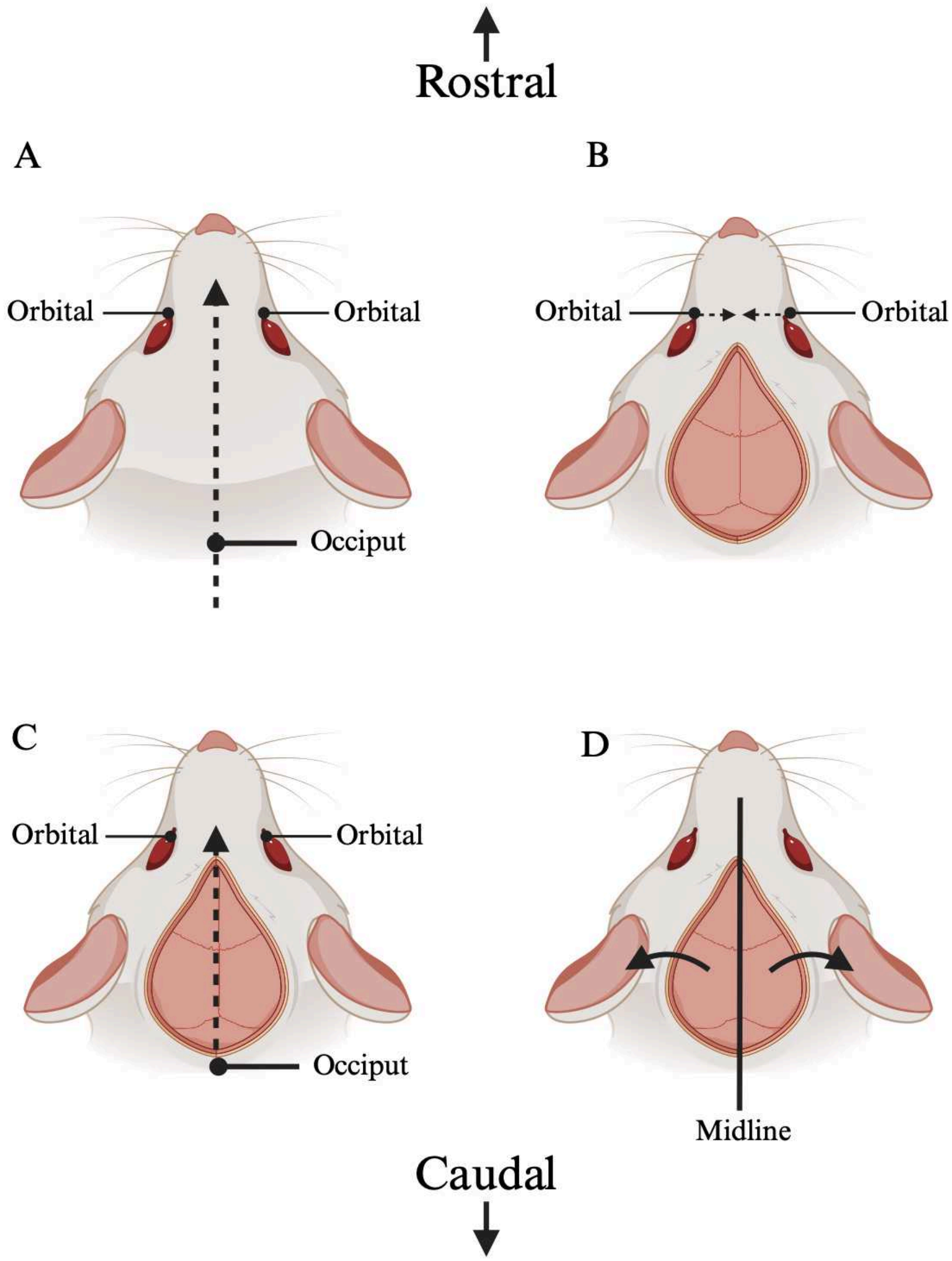

Fig 1. Brain Extraction. **[A]** Midline sagittal skin incision from occiput to rostrum. **[B]** Transverse incision between the orbitals. **[C]** Skull cut from occiput to orbit in a rostral direction. **[D]** Lateral separation of skull from brain. Created in BioRender.

5 Place the tips of the small scissors in the orbitals and cut the skull transversely as seen in **Fig 1B**.

6 Fixing the tips of the small scissors under the occiput of the skull, with slow rostral progression, cut on the midline with small precise movements to the previous orbit

incision, see **Fig. 1C.**

7 Placing the forceps under the skull at the incision on the left side, grip and pull the skull laterally, perpendicular to the midline, peeling the skull from the brain, see **Fig. 1D**.

8 If the skull breaks into pieces, remove each piece individually until complete hemisphere exposure is acheived.

9 Repeat steps 7 and 8 on the right hemisphere.

10 Once full brain exposure is accomplished, place the forceps beneath the brainstem and excise the brain by gently pulling superiorly and rostrally. *Inverting the mouse so that the dorsal surface of the cranium is closest to the workbench can be helpful, as it allows gravity to assist in brain extraction.*
