# Supplementary material for: Neurological tissue dissection techniques in mouse models for reproducible scientific results: Brain, spinal cord, cerebrospinal fluid, and sciatic nerve
Source: PLoS One. 2026 Oct 7;21(10):e0358771. doi: 10.1371/journal.pone.0358771 (PMC13645030; doi:10.1371/journal.pone.0358771)
Supplement: Supplementary file 3 [file pone.0358771.s003.pdf]

Aug 06, 2026 Version 2

Brain Microdissection in Mouse Models V.2

DOI
https://dx.doi.org/10.17504/protocols.io.36wgqxq1ylk5/v2

Lilia Crew[1], Alyssa Seerley[2], Serena McElroy[2], Andrea Grindeland Panter[1,2], Lilia Crew[1,2]

[1]Touro University College of Osteopathic Medicine, Great Falls, MT, United States;
[2]Weissman Hood Institute at Touro University, McLaughlin Research Institute, Great Falls, MT, United States

Lilia Crew: Co-first author;
Alyssa Seerley: Co-first author
Andrea Grindeland Panter: Senior author

Alyssa Seerley
Weissman Hood Institute at Touro University; McLaughlin Rese...

Create & collaborate more with a free account

Edit and publish protocols, collaborate in communities, share insights through comments, and track progress with run records.

Create free account

OPEN ACCESS

DOI: https://dx.doi.org/10.17504/protocols.io.36wgqxq1ylk5/v2

Protocol Citation:
Lilia Crew, Alyssa Seerley, Serena McElroy, Andrea Grindeland Panter, Lilia Crew 2026. Brain Microdissection in Mouse Models. **protocols.io**
https://dx.doi.org/10.17504/protocols.io.36wgqxq1ylk5/v2
Version created by
Alyssa Seerley

License: This is an open access protocol distributed under the terms of the **Creative Commons Attribution License**, which permits unrestricted use, distribution, and reproduction in any medium, provided the original author and source are credited

Protocol status: Working
We use this protocol and it's working

Created: August 06, 2026

Last Modified: August 06, 2026

Protocol Integer ID: 322108

Keywords: brain microdissection, mouse brain, mouse models, brain regions, brain microdissection in mouse model, mouse brain hemisphere, method for the microdissection, microdissection, subsequent analysis of specific brain region, specific brain region, mouse model, mouse

Funders Acknowledgements:
NIH General Medical Sciences
Grant ID: P20GM152335

Abstract

This protocol describes a method for the microdissection of a mouse brain hemisphere that allows for subsequent analysis of specific brain regions.

Materials

Microdissection microscope
Weigh boat
Scalpel
Forceps
Fine tip thumb forceps

Mouse Brain Microdissection

1

Note

The following procedure was performed under a microdissection microscope. Full dissection shown in **Fig 1**. See **Fig 2** for a size reference of the mouse brain hemisphere after separation.

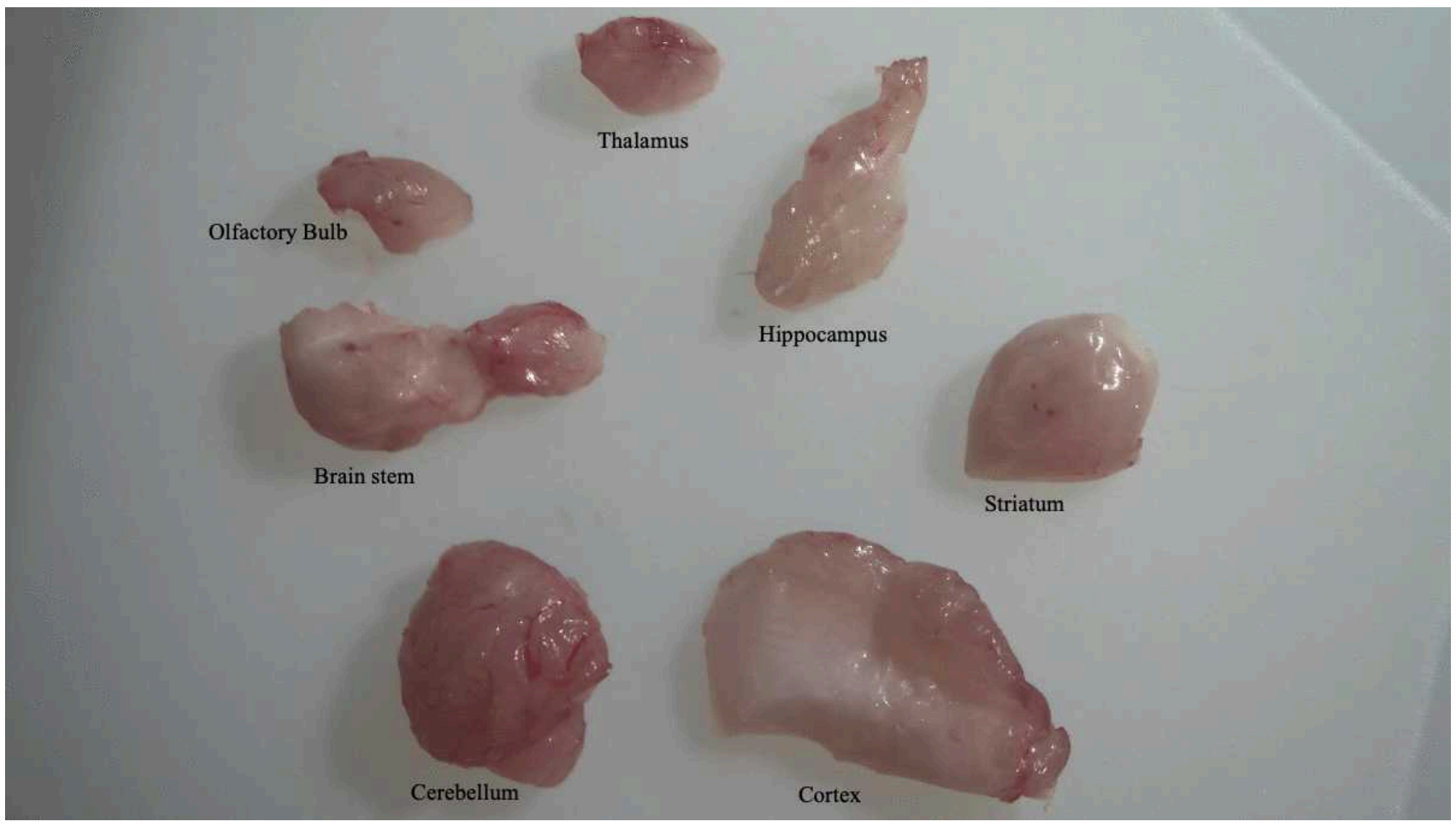

Fig 1. Completed Microdissection of the Mouse Brain. Labeled brain regions following careful dissection including the olfactory bulb, thalamus, hippocampus, striatum, brainstem, cerebellum, and cortex.

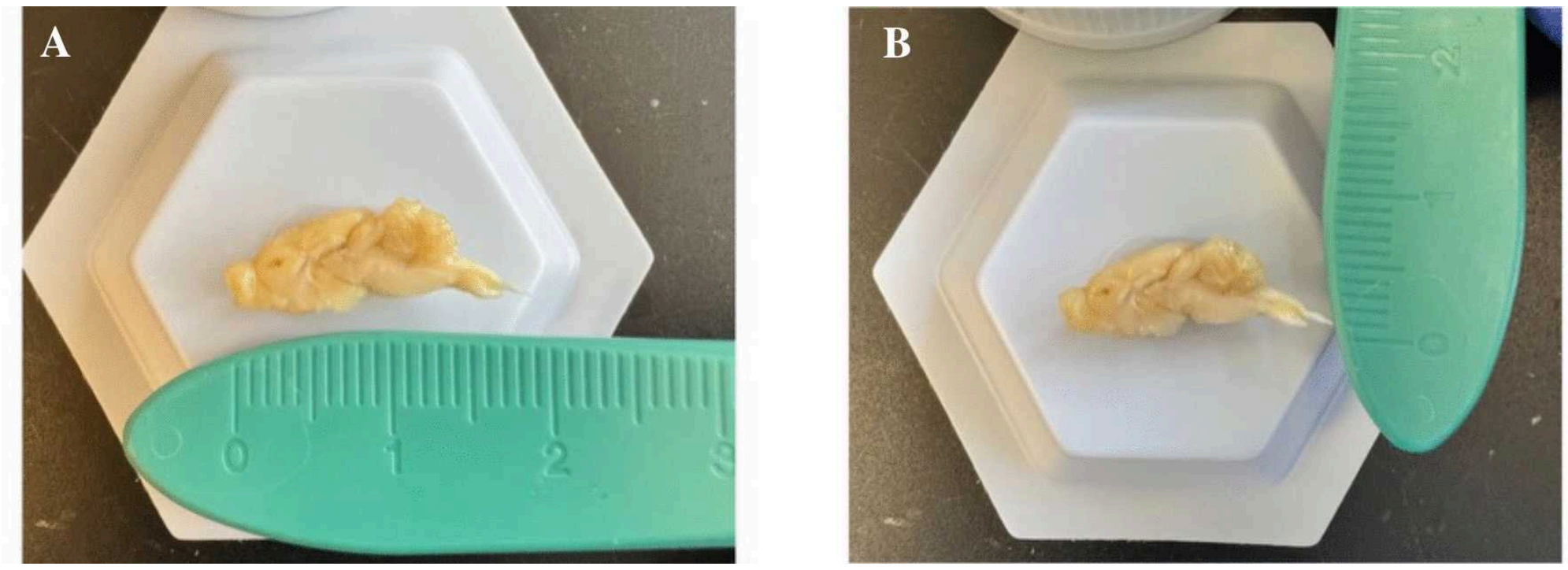

Figure 2. Size Reference of the Mouse Brain. Lateral-medial view of a fixed right hemisphere mouse brain. **[A]** Measuring approximately ~1.9cm in length and **[B]**~0.6cm in height.

2 Hemispheric Separation—Place the brain on a clean, preferably chilled, surface with the dorsal surface facing upward and the ventral surface facing downward **(Fig. 3A)**. Using a scalpel, make a sagittal cut along the longitudinal fissure to separate the cerebral hemispheres **(Fig. 3B)**. For this step and the subsequent dissections, a chilled work surface may be prepared by placing wet ice and one to two small pieces of dry ice in a lidded Petri dish."

3

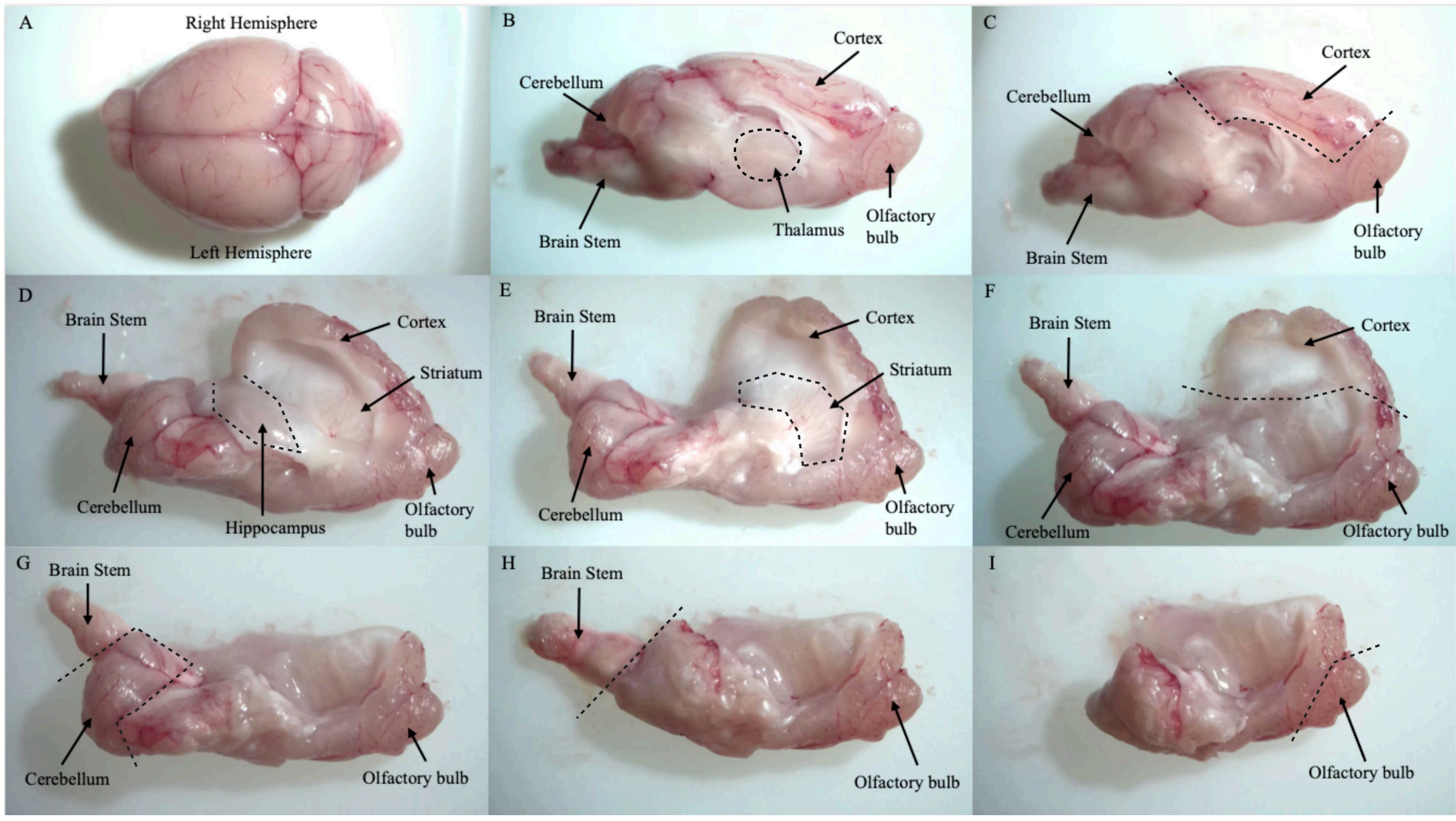

Fig 3. Brain Microdissection. Panel **[A]** shows the whole brain with labeled hemispheres. **[B]** sagittal view of left hemisphere **[C]** hemisphere without thalamus **[D]** cortex peeled back to reveal hippocampus and striatum **[E]** hemisphere without hippocampus, **[F]** hemisphere without striatum, **[G]** hemisphere without cortex, **[H]** hemisphere without cerebellum, **[I]** hemisphere without brain stem (Zeiss Stemi 508 & Axiocam 208 Color Photo). Created in BioRender.

4 *Thalamus* - On the medial side of the brain while holding the hemisphere steady with forceps, visualize and scoop out the thalamus with the scalpel using blunt dissection, paying attention to region boundaries, see **Fig 3C**.

5 *Hippocampus* - On the medial side of the brian, peel the cortex laterally, perpendicular to the midline using blunt dissection with the scalpel and while holding the hippocampus in place with the forceps to ensure separation of the two regions. Perform blunt dissection to extract the hippocampus medially toward the midline using the scalpel, see **Fig 3D and E**.

6 *Striatum* - Expose the striatum by removing the cortex and hippocampus as described above. Incise around the perimetere of the striatum with fine tip thumb forceps. Using the scalpel, scoop out the striatum with reference to natural tissue separtation, see **Fig 3F**. Striations help to differentiate the striatum from the cortex **Fig 3D**.

7 *Cortex* - Excise the base of the already dissected cortex using the scalpel blade, see **Fig 3G.**

8 *Cerebellum* - Remove the base of the cerebellum from the rest of the brain using a scalpel with attention to natural separation, see **Fig 3H**.

9 *Brainstem* - Excise the brainstem from the midbrain, see **Fig 3I.**

10 *Olfactory bulb* - Excise the olfactory bulb from the forebrain.
