# Supplementary material for: Neurological tissue dissection techniques in mouse models for reproducible scientific results: Brain, spinal cord, cerebrospinal fluid, and sciatic nerve
Source: PLoS One. 2026 Oct 7;21(10):e0358771. doi: 10.1371/journal.pone.0358771 (PMC13645030; doi:10.1371/journal.pone.0358771)
Supplement: Supplementary file 5 [file pone.0358771.s005.pdf]

Aug 06, 2026 Version 2

Mouse Spinal Cord Extrusion V.2

DOI
https://dx.doi.org/10.17504/protocols.io.dm6gp7181gzp/v2

Lilia Crew[1], Alyssa Seerley[2], Serena McElroy[2], Andrea Grindeland Panter[1,2], Lilia Crew[1,2]

[1]Touro University College of Osteopathic Medicine, Great Falls, MT, United States;
[2]Weissman Hood Institute at Touro University, McLaughlin Research Institute, Great Falls, MT, United States

Lilia Crew: Co-first author;
Alyssa Seerley: Co-first author
Andrea Grindeland Panter: Senior author

Alyssa Seerley
Weissman Hood Institute at Touro University; McLaughlin Rese...

Create & collaborate more with a free account

Edit and publish protocols, collaborate in communities, share insights through comments, and track progress with run records.

Create free account

OPEN ACCESS

DOI: https://dx.doi.org/10.17504/protocols.io.dm6gp7181gzp/v2

Protocol Citation:
Lilia Crew, Alyssa Seerley, Serena McElroy, Andrea Grindeland Panter, Lilia Crew 2026. Mouse Spinal Cord Extrusion. **protocols.io**
https://dx.doi.org/10.17504/protocols.io.dm6gp7181gzp/v2
Version created by
Alyssa Seerley

License: This is an open access protocol distributed under the terms of the **Creative Commons Attribution License**, which permits unrestricted use, distribution, and reproduction in any medium, provided the original author and source are credited

Protocol status: Working
We use this protocol and it's working

Created: August 05, 2026

Last Modified: August 06, 2026

Protocol Integer ID: 322079

Keywords: mouse spinal cord extrusion this protocol, mouse spinal cord extrusion, mouse spinal cord, hydropulsion technique, method for the extrusion, extrusion, technique, mouse

Funders Acknowledgements:
NIH General Medical Sciences
Grant ID: P20GM152335

Abstract

This protocol describes a method for the extrusion of a mouse spinal cord using the hydropulsion technique.

Materials

70% EtOH
Petri dish
1x Phosphate buffered saline (PBS)
Large scissors
Forceps
10ml syringe
Pipette tip

Mouse Spinal Cord Extrusion

1 Place mouse on ventrum and coat with EtOH.

2 Decapitate the mouse at the occiput.

3 Remove the skin covering the spine.

4 Perform a transverse scissor slice immediately cranial to the pelvis. This should extract the spine from the cervical vertebrae to the lumbosacral junction (Fig. 1A)

5

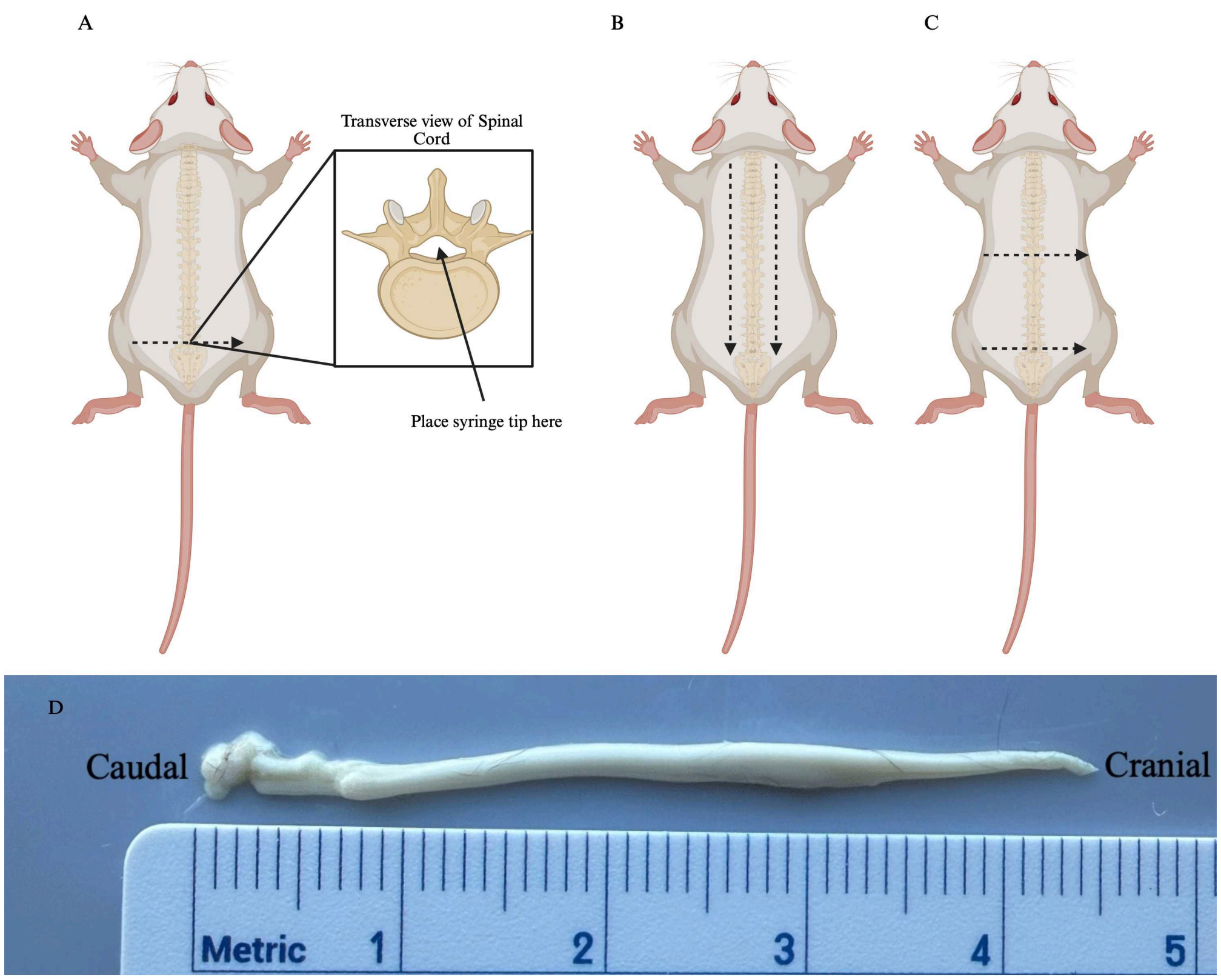

Fig 1. Spinal Cord Post-Extraction. [A] Transverse scissor slice cranial to the pelvis with a transverse view of the spinal cord; **[B]** Two slices lateral to the spinal vertebrae; **[C]** One slice at the thoraco-lumbar junction and one slice immediately cranial to the pelvis. **[D]** Whole spinal cord post-extraction. Created in BioRender. https://BioRender.com/4olnrjf

6 While holding the body, lift the cut end on the spinal cord so there is enough space to fit syringe tip to the spinal cord cavity. Ensure the spinal cord is visible and remove excess tissue if necessary (Fig. 1A).

7 Using a 10ml syringe filled with chilled (4 °C) phosphate buffered saline (PBS), secure a tight seal with the syringe (may need to add a pipette tip with the tip cut to fit the size of the spinal cord) to the spinal cord cavity, apply firm force to plunger allowing hydrostatic pressure to extrude the spinal cord into a petri dish filled with PBS(Fig. 1D).

8 The extrusion of the spinal cord may be easier if specific spinal regions are dissected. To do this, start at the cranial side of the body using the large scissors, slice through the tissues and ribs lateral to the spinal vertebrae progressing caudally to the pelvis (Fig. 1B). If not already done, perform a transverse scissor slice immediately cranial to the pelvis (Fig. 1C). Remove additional organs with froceps and scissors on the ventral side of the

spine. Next, complete a transverse scissor slice at the thoraco-lumbar junction (cut just below last rib) to isolate the thoracic spinal cord from the lumber spinal cord (Fig. 1C). Hold the spinal cord in a fashion that elimainates the curvature and proceed with extrusion. If the spinal cord is stuck to the end of the vertebrae, carefully use the forceps to manipulate the spinal cord out of the cavity.

9 Store as desired.
